# A poly(trisulfide) oligomer with antimicrobial activity

**DOI:** 10.1039/d5sc09816e

**Published:** 2026-04-16

**Authors:** Jasmine M. M. Pople, Ocean E. Clarke, Romy A. Dop, Thomas P. Nicholls, Harshal D. Patel, Witold M. Bloch, Zhongfan Jia, Sara J. Fraser-Miller, Evangeline C. Cowell, Jillian M. Carr, Daniel R. Neill, Joanne L. Fothergill, Bart A. Eijkelkamp, Tom Hasell, Justin M. Chalker

**Affiliations:** a College of Science and Engineering, Flinders University Bedford Park South Australia 5042 Australia jasmine.pople@flinders.edu.au bart.eijkelkamp@flinders.edu.au justin.chalker@flinders.edu.au; b Department of Clinical Infection, Microbiology and Immunology, Institute of Infection, Veterinary and Ecological Sciences, University of Liverpool Liverpool L69 7ZD UK; c Department of Chemistry, University of Liverpool Liverpool L69 7ZD UK T.Hasell@liverpool.ac.uk; d Flinders Microscopy and Microanalysis, College of Science and Engineering, Flinders University Bedford Park South Australia 5042 Australia; e Flinders Health and Medical Research Institute, College of Medicine and Public Health, Flinders University Bedford Park 5042 South Australia Australia; f Division of Molecular Microbiology, University of Dundee DD1 5EH Dow Street Dundee UK

## Abstract

Antimicrobial resistance is a growing threat to human health and agriculture. Sulfur-containing compounds and elemental sulfur have a long history as antimicrobials, but challenges related to solubility have limited their use. Recent advances in sulfur polymer chemistry have enabled the development of novel sulfur-rich materials with antimicrobial activity. However, most of these materials are water-insoluble, limiting their use in medicine and crop protection. Here, we report the synthesis of a linear poly(trisulfide) *via* photochemical ring-opening polymerization of a cyclic trisulfide monomer bearing a carboxylic acid. Deprotonation of the carboxylic acid renders the poly(trisulfide) water soluble, with concomitant chain scission *via* S–S cleavage. The resulting poly(trisulfide) oligomers exhibited potent antifungal activity against *Candida albicans* (CAF 2.1, MIC_90_ < 8 µg mL^−1^; SAH 1.1, MIC_90_ = 128 µg mL^−1^) and *Candida auris* (SAH 2.1, MIC_90_ = 128 µg mL^−1^). The poly(trisulfide) oligomers also exhibited antibacterial activity against *Staphylococcus aureus* (USA300, MIC_90_ = 16 µg mL^−1^; SH1000, MIC_50_ < 32 µg mL^−1^). In control experiments, the monomer alone had much lower antimicrobial activity against *C. albicans* and *S. aureus*. Toxicity assays of the poly(trisulfide) oligomer revealed it was not harmful to mammalian cells at these concentrations. The findings are a new direction for biological applications of sulfur polymers and a new strategy to support the battle against antimicrobial resistance.

## Abstract 2

A poly(trisulfide) was prepared by a photochemical ring-opening polymerization. The polymer was rendered water-soluble by treatment with base, yielding oligomeric products of chain scission active against fungal and bacterial pathogens.

## Graphical abstract

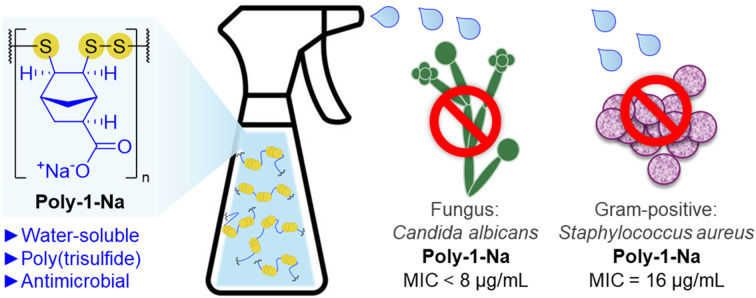

## Introduction

Antibacterial^1,2^ and antifungal^3–6^ resistance is a growing threat to human health and agriculture.^7^ To overcome these challenges in healthcare and food production, novel classes of antimicrobials and new modes of action are urgently required.^1–6^ Elemental sulfur has a long history as an antimicrobial in human medicine^8–11^ and crop protection.^12,13^ Likewise, organic polysulfanes^14^ such as the trisulfides found in plants of the *Allium* genus and other natural systems have been explored as antimicrobials.^15–17^ The activity of these sulfur-based treatments are a consequence of the diverse chemistry of sulfur and the S–S bond, including complex redox processes, reactions with enzymes, and generation of hydrogen sulfide and other bioactive metabolites.^12,16^ The success of sulfur-derived antimicrobials notwithstanding, there remain challenges in the formulation of water-insoluble inorganic sulfur^12^ and the complications associated with mixtures of volatile and malodorous polysulfides, such as those produced in alliums.^17^

One recent approach to making new and more readily processible formulations of sulfur-derived antimicrobials has been to make polymers from elemental sulfur.^18–20^ This approach has been driven by the widespread access to such polymers, made possible by invention of inverse vulcanization and related copolymerizations of elemental sulfur.^21,22^ The biological applications of these polymers are relatively new, but growing rapidly and include biodegradable fertilizers^23–26^ and antimicrobial surfaces,^18–20,27^ powders,^28^ coatings,^29^ adhesives,^30^ plastic additives,^31^ and nanocomposites.^32^ In these applications, the antimicrobial activity is encouraging but water insolubility makes precise formulation and dosing challenging for medical and agricultural applications. A notable exception is a recent report by Hasell and co-workers that showed a water soluble polymer made from sulfur and 1-allyl-3-vinylimidazolium chloride inhibited growth of *S. aureus*.^33^ Additionally, while there are a few reports of water-soluble polymers made by inverse vulcanization and related polymerizations,^33–37^ there is a need for more general and controlled methods for their synthesis.

In this paper, we report a photochemical ring-opening polymerization of a well-defined cyclic trisulfide bearing a carboxylic acid. The polymerization provides a poly(trisulfide) with well-defined structural units, and water solubility when the carboxylic acid is deprotonated. In the deprotonation, the base also causes chain scission through cleavage of S–S bonds. The resulting oligomers are the bioactive species investigated in this study.

The photo-polymerization method and its mechanism were recently reported as part of a larger effort to make a recyclable, water-insoluble gold sorbent.^38,39^ Here we show the method is applicable to the synthesis of a poly(trisulfide), that—when activated with base—generates water-soluble poly(trisulfide) oligomers that are effective growth inhibitors of the fungus *Candida* spp. and Gram-positive bacterium *S. aureus*. Interestingly, the monomer has a far lower activity against these pathogens. Encouragingly, the 50% cytotoxic concentration (CC_50_) values against the human hepatocellular carcinoma cell line (HepG2) were greater than the MICs against *Candida* spp. and *S. aureus* and caused no hemolysis of mammalian red blood cells at any concentration tested. This is an encouraging result that suggests selective anti-microbial action when applied between 8 and 128 µg mL^−1^, and no toxicity to liver and red blood cells at these concentrations. Overall, our approach provides a new pathway for antimicrobial development of sulfur-rich poly(trisulfide) polymers.

## Results and discussion

### Monomer and polymer synthesis

**Fig. 1:**
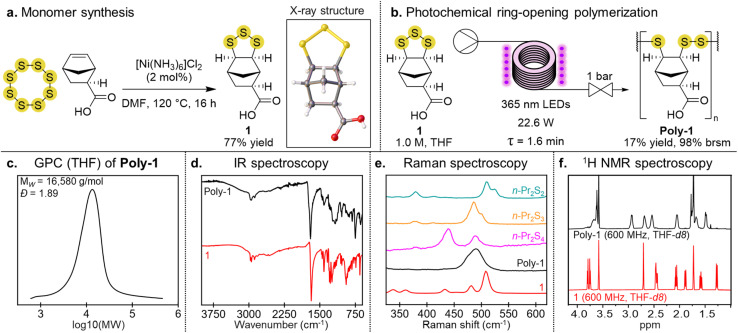
(a) Reaction of sulfur and *exo*-5-norbornenecarboxylic acid to form monomer 1. (b) Photochemical ring-opening polymerization provides linear poly(trisulfide) poly-1. (c) GPC trace of poly-1 (THF). (d) Infrared spectra of monomer 1 and poly-1. (e) Raman spectra of monomer 1, poly-1, *n*Pr_2_S_2_, *n*Pr_2_S_3_, and *n*Pr_2_S_4_. (f) ^1^H NMR spectra of monomer 1 and poly-1 (THF-*d*_8_).

Monomer 1 was first synthesized by the reaction of *exo*-5-norbornenecarboxylic acid with sulfur in the presence of a [Ni(NH_3_)_6_]Cl_2_ catalyst (Fig. 1a).^35,40^ The addition of sulfur occurred on the *exo* face, providing the cyclic trisulfide in 77% yield. The stereochemistry of 1 was confirmed by X-ray crystallography.^35^ To make poly-1, we used a continuous flow photopolymerization recently reported by our team.^38,39^ In this process, a 1.0 M solution of the monomer was pumped through the photoreactor with irradiation provided by LEDs (365 nm wavelength) and a residence time of 1.6 minutes (Fig. 1b). The reaction mixture was then collected and the solvent removed. The polymer was purified by redissolving the mixture in THF and then selectively precipitating with chloroform. Excess monomer remaining in solution could be recovered and re-used to make more polymer. The yield of the polymer was typically >90% based on recovered starting material. Under these conditions the weight average molecular weight (*M*_W_) of poly-1 was 16 580 g mol^−1^ with a dispersity of *Đ* = 1.89, as assessed by gel permeation chromatography (GPC) using THF as the eluent (Fig. 1c). The infrared spectrum of poly-1 had the expected carbonyl stretch at 1700 cm^−1^ (Fig. 1d). The Raman spectrum of poly-1 also indicated the polymer was predominantly a poly(trisulfide), based on its signal at 489 cm^−1^ and comparison to reference di, tri- and tetrasulfides (Fig. 1e and discussion below). The ^1^H NMR spectrum of poly-1 exhibited broad signals characteristic of a polymer structure (Fig. 1f). Several grams of poly-1 have been made by this photopolymerization,^38,39^ with good reproducibility (see SI page S17 for synthesis and characterization across several batches).

Mechanistically, the polymerization is initiated by photochemical cleavage of an S–S bond in the monomer. The resulting diradical can then propagate by reaction with another monomer, forming new S–S bonds in a ring-opening polymerization. Our previous studies revealed that disulfides and trisulfides are expected to form when the thiyl radical reacts, but trisulfide formation is favored kinetically when the perthiyl radical reacts.^38,39^ A consequence of this selectivity is that the polymer is predominately a poly(trisulfide). Indeed, Raman spectroscopy revealed the characteristic signal for linear trisulfides at 490 cm^−1^ (Fig. 1e). An important aspect of the photopolymerization is the use of flow chemistry and excess monomer. The short residence time ensures photochemical initiation, while reducing photochemical cleavage of the trisulfides in the polymer.^38,39^ Excess monomer is required, as this reversible polymerization is endergonic.^38,39^

**Fig. 2:**
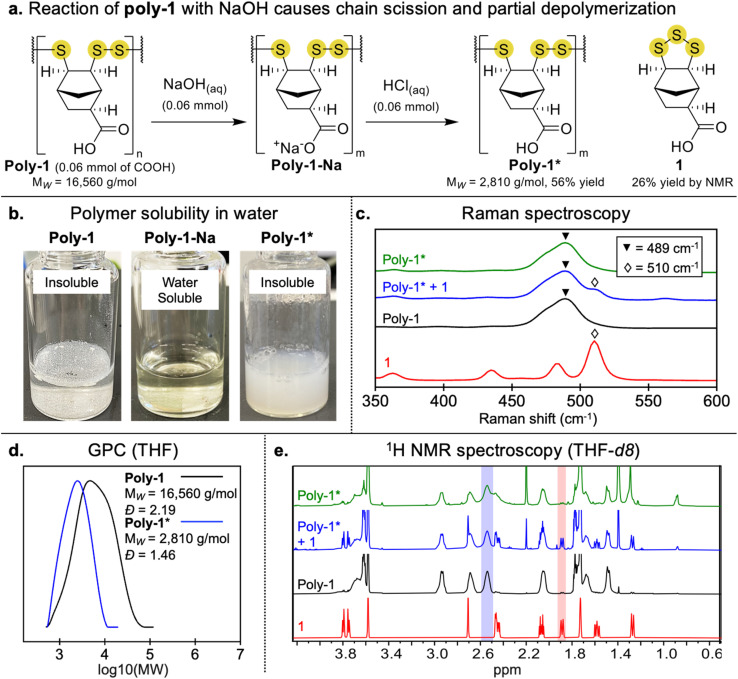
(a) Reaction of poly-1 with NaOH causes chain scission, and depolymerization. Product poly-1* (precipitated by the addition of HCl) is a poly(trisulfide) oligomer. (b) Digital photos of water-insoluble poly-1, the water-soluble poly-1-Na and regenerated monomer, and the insoluble poly-1* formed after addition of HCl. (c) Raman spectroscopy of monomer 1, poly-1, the mixture of the poly-1* oligomers and regenerated monomer formed from reaction of NaOH, and purified poly-1* oligomers. The key signals include the 510 cm^−1^ of the cyclic trisulfide in monomer 1 and the 489 cm^−1^ signal of the linear trisulfides present in poly-1 and the oligomeric poly(trisulfide) poly-1*. (d) GPC trace providing evidence of depolymerization when poly-1 reacts with NaOH. (e) ^1^H NMR spectra of monomer 1, poly-1, the mixture of the poly-1* oligomers and regenerated monomer formed from reaction of NaOH, and purified poly-1* oligomers. A signal unique to the poly(trisulfides) are highlighted in blue (present in both poly-1 and poly-1*). A signal unique to the monomer 1 is highlighted in red.

Next, poly-1 was deprotonated by reaction with sodium hydroxide (NaOH) to generate the sodium salt, poly-1-Na, which was fully soluble in water (Fig. 2a and b). The water solubility of poly-1-Na enabled quantification of nucleophilic end groups using Ellman's reagent (SI pages S18 and S19). It was found that poly-1-Na showed a significant reaction with Ellman's reagent and significantly more than the cyclic trisulfide, 1-Na. The response was quantified using a calibration curve from the reaction of glutathione and Ellman's reagent. On average, poly-1-Na contains an apparent thiol content of ∼1%. We tentatively attribute the reaction of Ellman's reagent and poly-1-Na to nucleophilic end groups such as thiols or perthiols, but we cannot rule out other nucleophilic species such as sulfinates. Higher oxidation species such as sulfonates may also be formed, which would not be detectable in this assay. We also cannot rule out the formation of cyclic polymers with no reactive end groups.

Because of the potential lability of the trisulfide groups, we wanted to assess if the reaction with NaOH caused any depolymerization or other S–S cleavage. We therefore converted poly-1-Na back to poly-1 by the addition of hydrochloric acid (HCl). Upon re-protonation, the precipitated product (poly-1*) was collected by centrifugation. Interestingly, GPC analysis of poly-1* in THF revealed a large decrease in *M*_W_ (16 560 g mol^−1^ for this batch of poly-1 to 2810 g mol^−1^ in the example shown in Fig. 2d). The decrease in molecular weight was likely due to cleavage of S–S bonds in the polymer backbone by the nucleophilic hydroxide ions, leading to smaller polymers, oligomers, and even regenerated monomer. Such cleavage of S–S bonds is known to occur in the presence of NaOH, which has been used to deliberately depolymerize poly(disulfides).^41^ The positive Ellman's test is also consistent with S–S cleavage and generation of nucleophilic end groups such as thiols or perthiols.

Despite the reduction in molecular weight, Raman spectroscopy of poly-1* indicated that major species were still oligomeric poly(trisulfides) (Fig. 2c). ^1^H NMR spectroscopy could also distinguish monomer and these oligomeric species in poly-1* (Fig. 2e). To quantify the relative amounts of poly(trisulfide) compared to regenerated monomer in the mixture, a partial least squares regression (PLSR) model was developed and applied (see SI pages S26–S28 for PLSR model development). The analysis predicted a poly(trisulfide) content of 77 ± 6% and includes all oligomers and shorter polymers linked by linear trisulfides, with the remainder of the mixture comprised of regenerated monomer 1. This analysis also confirms that some depolymerization and formation of 1-Na occurs when poly-1 is reacted with NaOH.

Investigating this reaction further, we found that excess NaOH (5 equivalents per repeating unit) caused rapid depolymerization and regeneration of monomer 1 in a 61% isolated yield (with identity of 1 confirmed by ^1^H NMR spectroscopy, melting point and high-resolution mass spectrometry, SI pages S20 and S21). To maintain the oligomeric structure and reduce the amount of regenerated monomer, poly-1-Na was subsequently made by carefully titrating in 1 equivalent of NaOH for each carboxylic acid in poly-1. Interestingly, it was found when two separate samples of poly-1 with different molecular weights (*M*_W_ = 25 580 g mol^−1^ and *M*_W_ = 11 250 g mol^−1^) were treated with NaOH (1 equivalent for each carboxylic acid), they converged to a similar molecular weight distribution (SI page S22). In another unexpected result in our studies of the chain scission in poly-1, we found that dissolving poly-1 in DMSO caused rapid depolymerization, with monomer 1 isolated in 56% yield (SI pages S23 and S24). We intended to use a DMSO solution of uncharged poly-1 in biological testing, but the depolymerization prevented such tests. More generally, this result highlights the reactivity of poly(trisulfides) and how base and solvents can cleave S–S bonds and cause depolymerization.

Overall, these experiments indicated that poly-1-Na was still a poly(trisulfide), but its molecular weight was an order of magnitude less than the originally synthesized poly-1. This is an important caveat in analysis of the antimicrobial activity of poly-1-Na, as it should be considered a dynamic and reactive system of oligomers that may generate reactive intermediates responsible for any bioactivity. (A summary of these outcomes and characterization data are provided in the SI pages S29–S31). Poly-1-Na was fully water-soluble which facilitated dosing in antimicrobial assays. The deprotonated monomer (1-Na) was also fully soluble in water and tested for antimicrobial activity so that its activity could be compared to its oligomeric form—an important control given the evidence for partial depolymerization of poly-1-Na. Sodium polyacrylate was also used as a sulfur-free control polymer bearing carboxylates.

## Antimicrobial assays

**Fig. 3:**
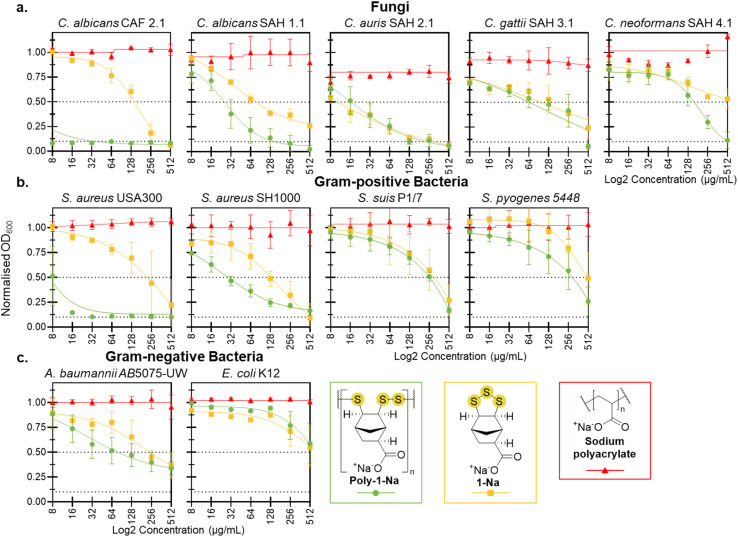
Activity assays against (a) fungi, (b) Gram-positive bacteria, and (c) Gram-negative bacteria. Normalized absorbance of cultures at 600 nm, in the presence of poly-1-Na, 1-Na, and sodium polyacrylate between 8 µg mL^−1^ and 512 µg mL^−1^ during a 20 hours incubation period at 37 °C. *C. albicans* (SAH 1.1), *C. auris* (SAH 2.1), *C. gattii* (SAH 3.1), and *C. neoformans* (SAH 4.1) were incubated for 48 hours. Data points represent the mean of three biological replicates (*n* = 3), and error bars indicate the standard error of the mean. The solid line corresponds to a non-linear regression fit (inhibitor *vs.* response, variable slope) performed in GraphPad Prism. Black dotted lines indicate the MIC_90_ and MIC_50_.

Antimicrobial activity of poly-1-Na, 1-Na, and sodium polyacrylate was assessed against model fungal species (*Candida* spp. and *Cryptococcus* spp.), Gram-positive bacteria (*Staphylococcus aureus* and *Streptococcus* spp.), and Gram-negative bacteria (*Escherichia coli* and *Acinetobacter baumannii*). Aqueous solutions of poly-1-Na, 1-Na, and sodium polyacrylate at several concentrations (0, 8, 16, 32, 64, 128, 256, and 512 µg mL^−1^) were supplemented to cultures of each pathogen. All cultures were then incubated at 37 °C for 20 hours except for *C. albicans* (SAH 1.1), *C. auris* (SAH 2.1), *C. gattii* (SAH 3.1), and *C. neoformans* (SAH 4.1), which required 48 hours of incubation to achieve robust growth (optical density at 600 nm [OD_600_] = >0.3). The OD_600_ was measured following incubation to determine the effect on total growth (Fig. 3). The derived MIC_90_ and MIC_50_ values, representing a 90% and 50% reduction in growth, respectively, demonstrated inhibition by poly-1-Na and 1-Na at 512 µg mL^−1^ or less for all microbes, with the exception of *C. neoformans* SAH 4.1 (1-Na only) and *E. coli* K12 (1-Na and poly-1-Na). The most effective antimicrobial activity was observed in the three *Candida* spp. studied here, all exhibiting MIC_90_ values of 512 µg mL^−1^ or lower.

### Antifungal activity

The antifungal activity was assessed against a reference strain of *C. albicans* (CAF 2.1), as well as clinical isolates of *C. albicans*, *C. auris*, *C. gattii*, and *C. neoformans* (Fig. 3a). These species are human opportunistic pathogens, causing life threatening infections in immunocompromised individuals.^42–44^*C. albicans*, *C. auris*, and *C. neoformans* represent three of the four highest-priority fungal pathogens identified by the World Health Organization, highlighting the urgent need for new therapeutic strategies to combat these pathogens.^45^*Candida* spp. is also difficult to treat due to increased resistance to antifungal drugs.^43,46^ Infections caused by *Cryptococcal* spp. result in >140 000 deaths annually, largely due to antifungal resistance and difficulty of treating infections in the central nervous system.^47^ It is therefore important to identify new antifungal agents to combat these infections, and our motivation to assess poly-1-Na for antifungal activity against these target fungal pathogens.

Poly-1-Na showed excellent growth inhibition of the reference strain *C. albicans* (CAF 2.1), with a MIC_90_ of <8 µg mL^−1^. Interestingly, the deprotonated monomer (1-Na) was significantly less active than its oligomeric form, with a MIC_90_ of 256 µg mL^−1^. The sodium polyacrylate control had no effect on *C. albicans* growth at any concentration tested. When poly-1-Na was assessed using a clinical isolate of *C. albicans* (SAH 1.1), an increase in the MIC_90_ to 128 µg mL^−1^ was observed, indicating the CAF 2.1 strain is more susceptible to poly-1-Na. Nevertheless, poly-1-Na showed a significant inhibition of the clinical isolate *C. albicans* (SAH 1.1), with a MIC_50_ of 32 µg mL^−1^. Additionally, poly-1-Na and 1-Na showed similar, but significant growth inhibition of *C. auris* with a MIC_50_ of 16 µg mL^−1^. Against *Cryptococcus* spp. poly-1-Na was less potent, with a MIC_50_ of 128 µg mL^−1^ for *C. gattii* and MIC_50_ of 256 µg mL^−1^ for *C. neoformans*.

The significant difference in activity between the monomer and polymer against *C. albicans* is interesting. As discussed above, poly-1 is a reactive species, with evidence of S–S bond cleavage when it is prepared as poly-1-Na. Future work will focus on understanding what specific intermediates are responsible for the diverse antifungal activities observed. For the purpose of this initial study, the important conclusion is that poly-1-Na is a potent antifungal and a new lead in the development of novel strategies for combatting *Candida* spp. infections.

### Antibacterial activity

Gram-positive bacteria (*S. aureus* and *Streptococcus* spp.) and Gram-negative bacteria (*E. coli* and *A. baumannii*) were used to evaluate the antibacterial activity of poly-1-Na. These species represent clinically significant pathogens with a documented increase in resistance to traditional antibiotics.^48–53^ Identifying new antibiotics and modes of action is therefore of immediate importance in combatting drug-resistant infections.

MIC assays were conducted to assess the activity of poly-1-Na, 1-Na, and the sodium polyacrylate control. Against *S. aureus* (USA300), poly-1-Na showed excellent activity, with a MIC_90_ of 16 µg mL^−1^, while against *S. aureus* (SH1000) it demonstrated lower but still promising activity with a MIC_50_ of 32 µg mL^−1^ (Fig. 3b). Similar to *C. albicans*, the monomer (1-Na) showed significantly lower activity against both *S. aureus* strains compared to the poly(trisulfide). In contrast, the polymer and monomer exhibited similar activity against *S. suis*, *S. pyogenese* and *A. baumannii* and low activity against *E. coli* (Fig. 3b and c). The sodium polyacrylate control showed no effect on the growth of these bacteria at any concentration tested.

The activity of poly-1-Na against *S. aureus* highlights its potential as a lead for the development of novel antibacterial treatments. While it is not as potent as critical medicines such as vancomycin (MIC < 4 µg mL^−1^ against *S. aureus*),^54^ it is notable that poly-1-Na still shows promising activity and can be prepared readily on a multi-gram scale. As resistance to vancomycin and other traditional antimicrobial treatments emerges, new entities such as poly-1-Na may be useful leads in developing next-generation antibiotics.^55^

### Synergy assays

To explore the mechanism of action of poly-1-Na against the most susceptible microbial species, *S. aureus* (SH1000) and *C. albicans* (SAH 1.1), we employed a series of co-treatment assays. We hypothesized that poly-1-Na may act through multiple pathways, given the role of sulfur in redox processes and metal binding, as well as the potential effects of the poly(trisulfide) structure on membranes. Synergy assays were therefore performed with dipyridyl (extracellular iron chelation), paraquat (intracellular superoxide production), and sodium dodecyl sulfate (SDS) (cell membrane stress) in combination with a sub-lethal concentration of poly-1-Na (16 µg mL^−1^) (SI page S32). Across the concentrations of SDS and Paraquat tested, growth of *S. aureus* and *C. albicans* remained unchanged in the presence or absence of poly-1-Na. The absence of additive antimicrobial activity suggests that these compounds may inhibit or dampen the activity of poly-1-Na. Against *S. aureus*, a slight additive effect was observed for dipyridyl at 400 µM in combination with poly-1-Na (16 µg mL^−1^). However, no synergistic interactions were identified for any of the compounds tested. Given that co-treatment with these reagents reduced the apparent activity of poly-1-Na, further studies were undertaken to probe its effects on *C. albicans* SAH 1.1 using microscopy and on *S. aureus* SH1000 using flow cytometry.

### Effect of poly-1-Na on *Candida albicans* morphology

**Fig. 4:**
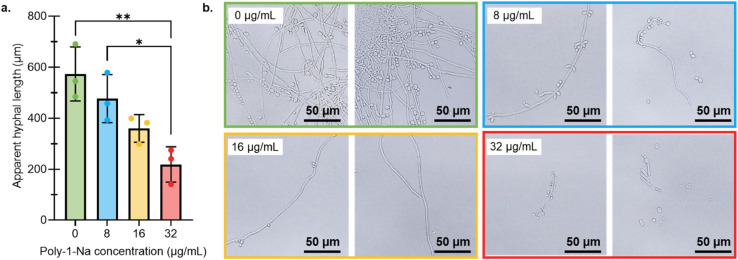
(a) Average hyphal length (µm) of *C. albicans* cultures grown in the presence of poly-1-Na (0, 8, 16, 32 µg mL^−1^) after a 24 hours incubation period at 37 °C. Data points represent the mean of three biological replicates (*n* = 3), and error bars indicate the standard error of the mean. Statistical analysis was done in GraphPad Prism using ordinary one-way ANOVA with multiple comparisons. (b) Microscopy images of *C. albicans* cultures grown in the presence of poly-1-Na (0, 8, 16, 32 µg mL^−1^) after a 24 hours incubation period at 37 °C. Images were taken with a 40× objective.

The hyphal length and the ratio of budding to non-budding yeast cells were analyzed for *C. albicans* SAH 1.1 cultured with sub-lethal concentrations of poly-1-Na (0, 8, 16, 32 µg mL^−1^). This experiment was designed to assess potential effects on cell morphology and replication modes. As shown previously, poly-1-Na decreased the overall cell density in a concentration-dependent manner (Fig. 3a). The ratio of budding to non-budding yeast cells showed no significant change across the concentrations tested (see SI pages S33–S35), while a statistically significant decrease in hyphal length was observed with increasing concentrations of poly-1-Na (Fig. 4a). These results suggest that, at the concentrations tested, poly-1-Na does not specifically affect budding-mediated replication of *C. albicans*, but that hyphal extension is sensitive to poly-1-Na. This is an important discovery because hyphal growth is associated with early biofilm formation and pathogenicity in *C. albicans*.^56^ In this context, the lack of an observable increase in hyphal formation with poly-1-Na may be of interest, although further studies are required to confirm morphology-specific effects.

### Effect of poly-1-Na on *Staphylococcus aureus* plasma membrane

**Fig. 5:**
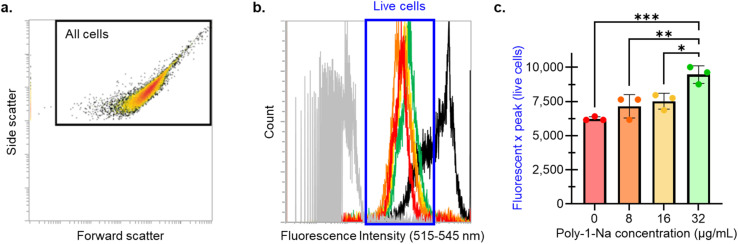
(a) Forward and side scatter plot of unstained *S. aureus* cells. The black box indicates the gate applied. (b) Fluorescence spectrum at 488 nm excitation and 515–545 nm emission of gated cells. Peaks from left to right are: unstained cells (no SYTOX, grey), 0 µg mL^−1^ of poly-1-Na (red), 8 µg mL^−1^ of poly-1-Na (orange), 16 µg mL^−1^ of poly-1-Na (yellow), 32 µg mL^−1^ of poly-1-Na (green), and dead cells (EtOH-treated, black). (c) Shift in fluorescent peak of live *S. aureus* cells in the presence of poly-1-Na (0, 8, 16, 32 µg mL^−1^). Data points represent the mean of three biological replicates, and error bars indicate the standard error of the mean. Statistical analyses were performed in GraphPad Prism using ordinary one-way ANOVA with multiple comparisons.

The effect of poly-1-Na on the permeability of the *S. aureus* SH1000 membrane was evaluated using SYTOX green staining and single-cell analysis by flow cytometry.^57^ A concentration-dependent increase in SYTOX fluorescence (Fig. 5) was revealed, consistent with increased membrane permeability. This shift suggests that sub-lethal concentrations of poly-1-Na can cause membrane disruption and provide preliminary insight into a possible mechanism behind the activity of poly-1-Na against *S. aureus*.

### Toxicity assays against mammalian cells

**Fig. 6:**
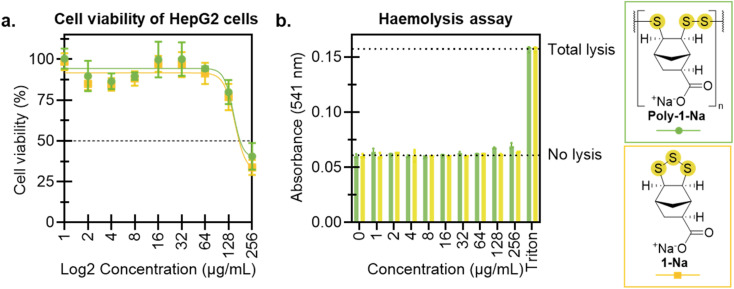
(a) HepG2 cell viability following exposure to poly-1-Na and 1-Na. Cells were cultured for 24 hours with increasing concentrations of poly-1-Na and 1-Na, then viability was quantitated by MTT assay. Values represent mean ± SD, *n* = 3. The 50% cytotoxic concentration (CC_50_) is indicated by the dotted line. (b) Haemolysis assay of rat red blood cells exposed to poly-1-Na and 1-Na for 30 minutes at 37 °C. 0.1% Triton-X was used as a positive control for total cell lysis. Values represent mean ± SD, *n* = 3.

To evaluate the potential of poly-1-Na as an antimicrobial, preliminary toxicity studies were conducted using human HepG2 cells and rat red blood cells. An MTT assay was carried out after exposing HepG2 cells to concentrations of poly-1-Na and 1-Na spanning 1–256 µg mL^−1^. For poly-1-Na, a reduction of cell viability was observed for doses of 128 and 256 µg mL^−1^, with a calculated 50% cytotoxic concentration (CC_50_) value of 147 µg mL^−1^ (Fig. 6a). The monomer (1-Na) showed a similar trend, with a CC_50_ of 153 µg mL^−1^. The comparable activity of the polymer and monomer suggests that the observed cytotoxicity may arise from extracellular or cell surface activities, given that poly-1-Na is likely too large to readily enter cells. However, it is possible that poly-1-Na breaks down into smaller reactive species that can penetrate cells and contribute to the observed activity. Additionally, neither poly-1-Na nor 1-Na induced haemolysis of red blood cells at any concentration tested (up to 256 µg mL^−1^) (Fig. 6b). These results are encouraging, as they indicate that poly-1-Na exhibits antimicrobial activity against target pathogens *C. albicans* and *S. aureus* while maintaining relatively low toxicity towards mammalian cells.

## Conclusions and outlook

A poly(trisulfide) bearing carboxylic acid groups (poly-1) was prepared using a newly developed photochemical ring-opening polymerization. Deprotonation with NaOH resulted in concomitant S–S chain scission, producing the water-soluble oligomeric species poly-1-Na. The antimicrobial activity of poly-1-Na was tested against a library of fungi and Gram-negative and Gram-positive bacteria. Encouraging activity was discovered against *C. albicans* (CAF 2.1, MIC_90_ < 8 µg mL; SAH 1.1, MIC_90_ = 128 µg mL^−1^), *C. auris* (SAH 2.1, MIC_90_ = 128 µg mL^−1^) and *S. aureus* (USA300, MIC_90_ = 16 µg mL; SH1000, MIC_50_ < 32 µg mL^−1^). Importantly, poly-1-Na did not exhibit toxic effects to human hepatocytes and rat red blood cells at these concentrations. Interestingly, the cyclic trisulfide monomer was less effective against *C. albicans* and *S. aureus* than the poly(trisulfide). This is an interesting result, because depolymerization was demonstrated by the reaction of poly-1 with base or DMSO. Such a capability could enable slow-release approaches or deactivation of the antimicrobial in a controlled way.

Future work will involve further investigating the mode of action to understand how poly-1-Na interacts with the microbes and inhibits their growth. As the polymer contains reactive sulfur species, we will explore its potential role in perturbing redox homeostasis, reactions with enzymes, and metal binding. Preliminary experiments reported here indicated potential membrane disruption of *S. aureus* and disruption of hyphal growth in *C. albicans*. More generally, this study highlights the potential for sulfur-derived polymers as novel agents to combat pathogenic fungi and bacteria. The abundance of sulfur and the simplicity and scalability of the polymerization reported here bode well for the future development of novel and readily available antimicrobial agents. Recent reports of new methods to make poly(trisulfides) will further support biological applications of this interesting class of polymers.^35,38,39,58,59^

## Author contributions

J. M. M. P., B. A. E., T. H. and J. M. C. conceptualized and designed the study and experiments. J. M. M. P., O. E. C. and R. A. D. performed the initial biological evaluations with supervision and input from D. R. N., J. L. F., T. H. and J. M. C.; J. M. M. P. and T. P. N. developed the polymerization methods and carried out chemical characterization. W. M. B. contributed to mechanistic and structural analysis. Z. J. contributed to material design, characterization, and mechanistic analysis. H. D. P. synthesized standards for Raman spectroscopy and S. J. F. M. carried out Raman spectroscopy and regression analysis. E. C. C. and J. M. C. carried out toxicity assays. J. M. M. P. led the mechanistic experimentation. J. M. M. P. and J. M. C. wrote the manuscript and all authors contributed to review and revision of the paper.

## Conflicts of interest

The authors have no conflicts of interest to declare.

## Supplementary Material

SC-OLF-D5SC09816E-s001

## Data Availability

Underlying data for biological testing is tabulated in the supplementary information (SI). Requests for all other data or materials can be sent to justin.chalker@flinders.edu.au. Supplementary information: full experimental details and additional chemical and biological characterization data for monomer 1, poly-1, and poly-1*. See DOI: https://doi.org/10.1039/d5sc09816e.
