# Supplementary material for: A poly(trisulfide) oligomer with antimicrobial activity
Source: Chem Sci. 2026 Apr 16;17(22):11017–26. doi: 10.1039/d5sc09816e (PMC13107205; doi:10.1039/d5sc09816e)
Supplement: SC-017-D5SC09816E-s001 [file SC-017-D5SC09816E-s001.pdf]

Supplementary Information (SI) for Chemical Science.
This journal is © The Royal Society of Chemistry 2026

Supporting Information
A poly(trisulfide) oligomer with antimicrobial activity

Authors: Jasmine M. M. Pople,[1†*] Ocean E. Clarke,[2†] Romy A. Dop,[2,3] Thomas P. Nicholls,[1] Harshal D. Patel,[1] Witold M. Bloch,[1] Zhongfan Jia,[1] Sara J. Fraser-Miller,[1,4] Evangeline C. Cowell,[5] Jillian M. Carr,[5] Daniel R. Neill,[6] Joanne L. Fothergill,[2] Bart A. Eijkelkamp,[1*] Tom Hasell,[3*] and Justin M. Chalker[1*]

1 College of Science and Engineering, Flinders University, Bedford Park, South Australia 5042, Australia

2 Department of Clinical Infection, Microbiology and Immunology, Institute of Infection, Veterinary and Ecological Sciences, University of Liverpool, Liverpool L69 7ZD, United Kingdom

3 Department of Chemistry, University of Liverpool, Liverpool L69 7ZD, United Kingdom

4 Flinders Microscopy and Microanalysis, College of Science and Engineering, Flinders University, Bedford Park, South Australia 5042, Australia

5 Flinders Health and Medical Research Institute, College of Medicine and Public Health, Flinders University, Bedford Park 5042, South Australia, Australia

6 Division of Molecular Microbiology, University of Dundee DD1 5EH, Dow Street, Dundee, United Kingdom

Contents:

Materials and methods S2
Characterization data for monomer **1** S6
^{1}H NMR spectrum of **1-Na** S12
Characterization data for **poly-1** S13
Batch-to-batch reproducibility of **poly-1** synthesis S17
Quantification of apparent thiol and perthiol end groups using Ellman's Reagent S18
Depolymerization of **poly-1** using excess NaOH S20
Preparation of **poly-1-Na** using two different molecular weights of **poly-1** S22
Depolymerization of **poly-1** by reaction with DMSO S23
Quantitative PLSR analysis by Raman spectroscopy for characterization of **poly-1*** S26
Summary of reaction of **poly-1** with **NaOH** (1 equivalent per carboxylic acid) S29
Synergy assays S32
Effect of **poly-1-Na** on *Candida albicans* morphology S33
Effect of **poly-1-Na** on *Staphylococcus aureus* plasma membrane S36
Toxicity assay and assessment of HepG2 cell viability S38
References S40

Materials and Methods

Reagents: The following materials were purchased and used as received: sulfur (Merck), exo-5-norbornenecarboxylic acid (Merck), Na_2SO_4 (Chem Supply), silica gel (40-63 µm particle size, Merck), chloroform-*d* (Cambridge Isotope Laboratories), THF-*d8* (Merk), D_2O (Merk), DMSO-*d6* (Merck), sodium polyacrylate (Sigma-Aldrich), sodium hydroxide (Sigma-Aldrich), L-glutathione (Sigma-Aldrich), Ellman's Reagent (Sigma-Aldrich) 2,2'-bypyridyl (dipyridyl) (Fluka Analytical), methyl viologen dichloride hydrate (paraquat) (Sigma-Aldrich), Sodium dodecyl sulphate (SDS) (VMR chemicals), SYTOX green (30 µM solution in DMSO, ThermoFisher Scientific). Solvents were purchased from either Chem Supply or RCI Labscan and were all analytical grade. Di-*n*-propyl disulfide (n-Pr_2S_2) and di-*n*-propyl trisulfide (n-Pr_2S_3) were purchased from Aaron Chemicals and used as received. Di-*n*-propyl tetrasulfide was synthesized following literature procedures.[1] Syringe filters used were purchased from Membrane Solutions (PTFE, 0.45 µm pore size, 25 mm diameter). Lysogeny Broth (LB), LB agar, Mueller Hinton (MHI) Broth, and phosphate buffered saline (PBS) were purchased from Sigma-Aldrich. Methicillin-resistant *S. aureus* strain USA300,[2] *E. coli* strain K12, and *C. albicans* strain CAF 2.1[3] were cultured from frozen stocks stored at the University of Liverpool. *S. aureus* stain SH1000, *S. suis* strain P1/7, *S. pyogenes* strain 5448, and *A. baumannii* strain AB5075-UW were cultured from frozen stocks stored at Flinders University. *C. albicans* strain SAH 1.1 is a clinical isolate obtained from SA Health and originally isolated from a blood culture. *C. auris* strain SAH 2.1 is a clinical isolate obtained from SA Health. *C. gattii* strain SAH 3.1 is a clinical isolate obtained from SA Health and originally isolated from cerebrospinal fluid. *C. neoformans* strain SAH 4.1 is a clinical isolate obtained from SA Health and originally isolated from lung tissue. The MTT Assay kit was purchased from Abcam, with additional growth media and antibiotics purchased from Gibco (see supporting information for more details).

Equipment:

Continuous flow photoreactor: A Vapourtec R-Series system with R2 C Plus pumping module was used to pump solutions through a coil of perfluoroalkoxyalkanes (PFA) tubing (10 mL). The coil was fitted to a Uniqsis Polar Bear Plus Flow which was used to control the temperature of the reaction mixture. A Uniqsis PhotoSyn provided LED irradiation at 365 nm (22.6 W). The back pressure of the system was controlled using a Vapourtec SF-10 reagent pump operating in pressure controller mode.

Gel permeation chromatography (GPC): Samples were dissolved in tetrahydrofuran (THF) and filtered using a 0.45 µm polytetrafluoroethylene (PTFE) syringe filter. Analysis of the molecular weight distributions of the polymers was performed using a Waters 2695 separations module, using two Agilent columns (PLgel MiniMix-C (5 µm)) and a matching Agilent guard column (MiniMix-C) and a Waters 410 refractive index detector. All samples were eluted in THF at a flow rate of 0.3 mL/min. Calibration was performed using narrow distribution polystyrene standards ($Đ$ <1.1) ranging from 500 to 2 million g/mol.

Nuclear magnetic resonance (NMR) spectroscopy: ^1H and ^{13}C NMR spectra were recorded on a Bruker Ultrashield Plus 600 MHz spectrometer at 600 MHz and 150 MHz, respectively. All spectra were recorded at 298 K. Deuterated solvents were used as solvent

and internal lock. Residual solvent peaks were used as an internal reference for $CDCl_3$ (^{1}H NMR δ 7.26 ppm; ^{13}C NMR δ 77.16 ppm), DMSO-*d6* (^{1}H NMR δ 2.50 ppm), D_2O (^{1}H NMR δ 4.9 ppm), and THF-*d8* (^{1}H NMR δ 3.58 ppm; ^{13}C NMR δ 67.21 ppm). Coupling constants (*J*) are quoted to the nearest 0.1 Hz. The following abbreviations, or combinations thereof, were used to describe NMR multiplicities: s = singlet, d = doublet, t = triplet, m = multiplet, ap. = apparent).

Infrared (IR) spectroscopy: IR spectra were recorded using a FTIR Perkin Elmer Frontier spectrometer between 4000 and 500 cm^{-1}. Samples were analyzed on a Perkin Elmer Universal ATR Sampling Accessory.

Raman spectroscopy: Raman spectra were collected using a Witec alpha300RAS+ Raman microscope at an excitation laser wavelength of 785 nm with a 10X objective to give a sample spot size of ~ 1.3 μm Ø. Six to 12 spectra were collected from different positions on each sample using project six plus software (WiTec Ulm, Germany) with a laser power of 80 mW, an integration time of 0.5 s and 60 co-additions. The Raman shift was calibrated to the 520.6 cm^{-1} peak for silicon.

High-resolution mass spectrometry (HRMS): HRMS were recorded on an ABSciex TripleTOF 5600+ and were reported as the observed molecular ion in negative mode.

Chemical synthesis:

*Monomer **1** synthesis:* The following method was adapted from the literature.[4,5] To a 100 mL round bottom flask equipped with a stir bar, *exo*-5-norbornenecarboxylic acid (1.00 g, 7.2 mmol) and DMF (10 mL) were added. Elemental sulfur (670 mg, 2.7 mmol, S_8) and $[Ni(NH_3)_6]Cl_2$ (31 mg, 0.1 mmol) were then added, and the resulting reaction mixture was heated to 120 °C. After 16 hours, the mixture was allowed to cool to room temperature before the solids were removed by filtration over a thin layer (~1 cm) of silica gel. The silica gel was washed with ethyl acetate (3 x 20 mL). Deionized water (20 mL) was added to the filtrate, the layers were separated, and the organic fraction was washed with deionized water (2 x 20 mL). The organic fraction was collected, dried (Na_2SO_4), filtered, and concentrated under reduced pressure. Purification by flash chromatography over silica gel (hexane:ethyl acetate (9:1)) delivered monomer **1** as a pale-yellow solid (1.3 g, 77% yield). Characterization data was consistent with that previously reported, with the structure and stereochemistry previously established by X-ray crystallography.[18b]

***Poly-1** synthesis:* A solution of **1** (4 mmol, 1.0 M) in THF (4 mL) was prepared. The solution was pumped through perfluoroalkoxyalkanes (PFA) tubing (10 mL, 21 °C) at a flow rate of 6 mL/min resulting in a residence time of 1.6 minutes with back pressure regulation at 1 bar. The monomer solution was irradiated continuously by light emitting diodes (LEDs) (365 nm, 22.6 W) in the flow reactor. The outflow of the reactor was collected in a round bottom flask. The solvent was removed under reduced pressure, and the resulting residue was redissolved in THF (2 mL) and then precipitated by adding chloroform (20 mL). The precipitated polymer was collected by filtration and redissolved in THF (2 mL) before precipitation into chloroform (20 mL). This process was repeated three times to purify the polymer from unreacted monomer. The resulting purified polymer (158 mg, 17% yield,

98% yield based on recovered **1**) was analyzed by ^{1}H NMR spectroscopy (THF-*d8*), IR spectroscopy, Raman spectroscopy and GPC (THF). The combined filtrate was concentrated under reduced pressure and analyzed by ^{1}H NMR ($CDCl_3$) spectroscopy to recover pure monomer **1** (768 mg, 81% recovery).

***Poly-1-Na** and **1-Na** preparation for MIC experiments:* **Poly-1-Na** and **1-Na** stock solutions (1024 µg/mL) were prepared by charging a glass vial with **poly-1** or monomer **1** (20 mg, 0.085 mmol of carboxylic acid groups) and sterile H_2O (19.41 mL). An aqueous solution of NaOH (590 µL, 0.145 M, 0.085 mmol) was then added and each solution was vortexed for 1 minute before being sterilised through filtration (0.45 µm). This concentration of NaOH is equimolar to the amount of carboxylic acids in **poly-1** or monomer **1**. Therefore, there is no excess NaOH in either the **poly-1-Na** or **1-Na** solution used for bioassays.

*Reaction of **poly-1** with NaOH (1 equivalent per carboxylic acid) and analysis of chain scission products*: A glass vial (20 mL) was charged with **poly-1** (15.0 mg, 0.06 mmol of carboxylic acid groups, M_W = 16,560 g/mol, $Đ$ = 2.19) and deionized water (2 mL). A NaOH stock solution (64 µL, 1.0 M, 0.06 mmol) was added and the mixture was vortexed for 1 minute to afford a homogenous solution. After 1 minute, HCl (22 µL, 3 M in H_2O, 0.06 mmol) was added causing the solution to turn cloudy due to product precipitation. The suspension was transferred to four 2 mL Eppendorf tubes and centrifuged (2 min, small benchtop centrifuge), forming a pellet. The supernatant was carefully decanted. The original vial was rinsed with deionized water (5 mL), and this solution was added to the Eppendorf tubes. The samples were vortexed and centrifuged again (2 minutes, small benchtop centrifuge) before decanting the supernatant. This washing step was repeated a third time. The resulting pellets were redissolved in a minimum amount of THF and transferred to a pre-weighed vial. After removal of the solvent under reduced pressure, 12.8 mg of the sample was recovered (85% mass recovery). The recovered sample (**poly-1***) was analysed by Raman spectroscopy, ^{1}H NMR spectroscopy (THF-*d8*), and GPC (THF). **Poly-1*** was then concentrated under reduced pressure and washed with chloroform (3 x 10 mL) to selectively remove regenerated monomer. After the chloroform wash, **poly-1*** (8.4 mg) was analysed by Raman spectroscopy and ^{1}H NMR spectroscopy (THF-*d8*).

Bacteria preparation, storage, and enumeration:
Glycerol stocks of *S. aureus* USA300, *E. coli* K12, and *C. albicans* CAF 2.1, *S. aureus* SH1000, *S. suis* P1/7, *S. pyogenes* 5448, *A. baumannii* AB5075-UW, *C. albicans* SAH 1.1, *C. auris* SAH 2.1, *C. gattii* SAH 3.1, *C. neoformans* SAH 4.1 were stored at −80 °C in 15% glycerol for long-term storage. For experimental use, frozen glycerol stocks were defrosted and streaked onto Lysogeny Broth (LB) agar plates, which were then incubated statically overnight at 37 °C. Bacterial cultures were prepared by swabbing 3-5 colonies into 5 mL of LB broth, followed by overnight incubation at 37 °C with agitation. Colony forming units (CFUs) were enumerated for *S. aureus* USA300, *E. coli* K12, and *C. albicans* CAF 2.1 by serially diluting the cultures in phosphate buffered saline (PBS) onto LB agar, using the Miles and Misra method.[6,7] CFU/cm^2 and CFU/mL were calculated using the following equation: CFU/mL = (No. of colonies × total dilution factor)/volume of culture plated in mL.

Determination of minimum inhibitory concentration:
Minimum inhibitory concentrations (MICs) were determined using a 96-well plate and were assessed according to the European Committee on Antimicrobial Susceptibility Testing (EUCAST) guidelines. An incubation period of 20 hours was used against *S. aureus* USA300, *E. coli* K12, *S. aureus* SH1000, *S. suis* P1/7, *S. pyogenes* 5448, and *A. baumannii* AB5075-UW in MH medium[8] and *C. albicans* CAF 2.1, in LB medium. An incubation period of 48 hours was used against *C. albicans* SAH 1.1, *C. auris* SAH 2.1, *C. gattii* SAH 3.1, and*C. neoformans* SAH 4.1 in LB medium. An initial OD_{600} of 0.1 (~5 × 10^5 CFU/mL) was used for the cell cultures prior to incubation. The OD_{600} was measured using a FLUOstar Omega microplate reader. Data were analysed in GraphPad Prism (10.4.1) with a nonlinear regression to fit a dose-response curve. Data represents mean ± SD of n = 3 biological replicates in each assay.

MTT assay for toxicity against human hepatocellular carcinoma cells (HepG2):
HepG2 cells were maintained in MEM (Gibco, cat. 11095080) supplemented with 10% FBS (Gibco, cat. 10099141), 100 units/mL Penicillin/100 µg/mL Streptomycin (Gibco, cat. 15140122), 1X GlutaMAX (Gibco, cat. 35050061). Cells were seeded at approximately 10^4 cells/well in a 96-well plate. The following day the test compound was added into the culture media at the relevant concentrations and cells cultured for 24 hrs. Brightfield images were obtained at 20x magnification using ZEISS Primovert microscope equipped with ZEISS Axiocam 208 color camera using the Labscope software. An MTT Assay (Abcam, ab211091) was performed to assess the cell viability/cytotoxicity. Accordingly, the growth media was removed and discarded, and then 50 µL of serum free media and 50 µL of the MTT solution were added to each well and incubated at 37°C for 3 hours. 150 µL of the MTT solvent was added to each well and incubated at room temperature, in the dark, with shaking for 15 minutes. Any precipitate formed was resuspended by pipetting and absorbance at 590 nm was then measured (SpectraMax iD5). Assay results were independently replicated (n = 2). Controls included no drug (100% viability), 0.2% Triton X-100 treated (0% viability) and media alone (background absorbance). Corrected absorbance readings were calculated for each of the replicate wells using the mean absorbance of the culture medium. Cell viability was calculated as follows

$$Cell\ viability\ (\%) = \frac{A_{sample\ corrected}}{A_{control\ corrected}} * 100$$

Data were analysed in GraphPad Prism (10.4.1) with a nonlinear regression to fit a dose-response curve. Data represents mean ± SD of n = 3 technical replicates in each assay.

Haemolysis assay: Haemolysis assay was performed to observe lytic properties of the **Poly-1-Na** compound, **1-Na** was used as a comparator, with no drug as a negative control and 0.1% Triton-X as a positive control for total cell lysis. 50 µL of a 2% rat red blood cell solution (obtained with approval for the use of scavenge rat tissue, ID 4198, Flinders University Animal Welfare Committee) was combined with 100 µL of the drug compound to make a final compound concentration of 1, 2, 4, 8, 16, 32, 64, 128, or 256 µg/mL respectively. This mixture was incubated at 37°C for 30 mins. Samples were then centrifuged at 2,500 x *g* for 6 min to pellet whole cells. 75 µL of resulting supernatant was transferred into the well of a 96-well plate. The absorbance was then measured at 541 nm using the SpectraMax iD5 plate reader. Data represents mean ± SD of n = 3 biological replicates in each assay.

Monomer 1 characterization

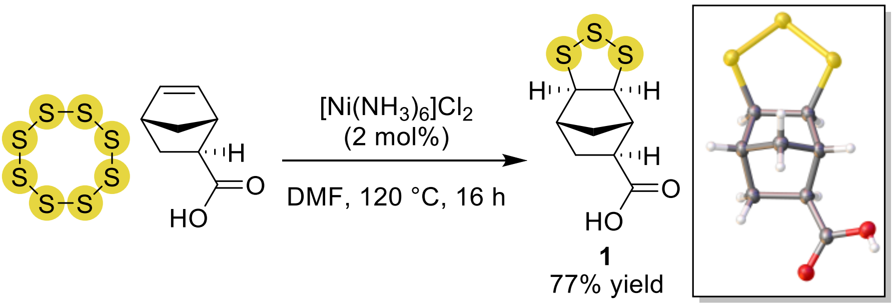

Reaction scheme and X-ray crystal structure of monomer **1**.[5] Melting point of **1**: 139-141 °C

^{1}H NMR spectrum (600 MHz, $CDCl_3$) of monomer **1**.

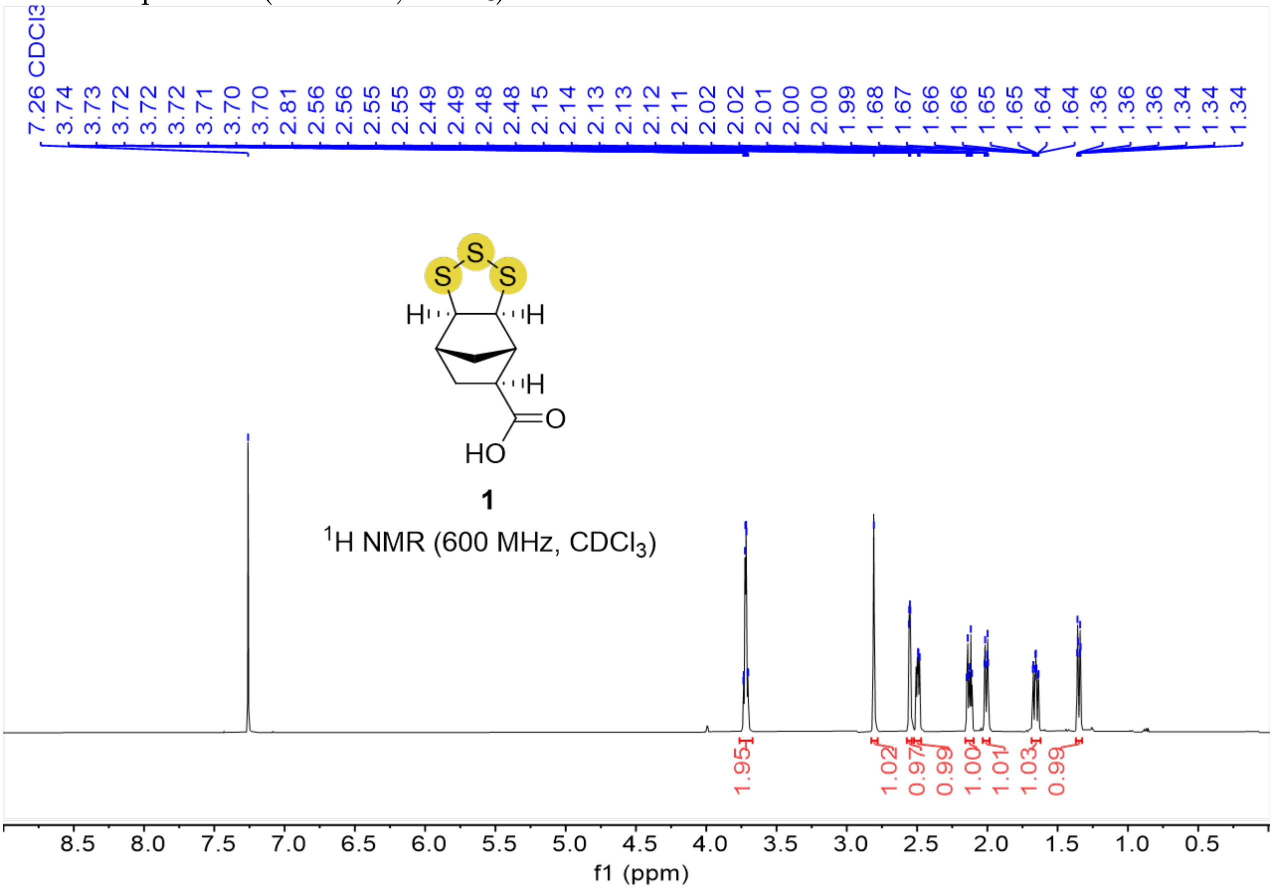

^{1}H NMR spectrum (600 MHz, $CDCl_3$) of monomer **1**. ^{1}H NMR (600 MHz, $CDCl_3$): δ 3.74-3.69 (m, 2H), 2.81 (ap. s, 1H), 2.56-2.54 (m, 1H), 2.51-2.47 (m, 1H), 2.15-2.10 (m, 1H), 2.03-1.98 (m, 1H), 1.68-1.62 (m, 1H), 1.36-1.32 (m, 1H).

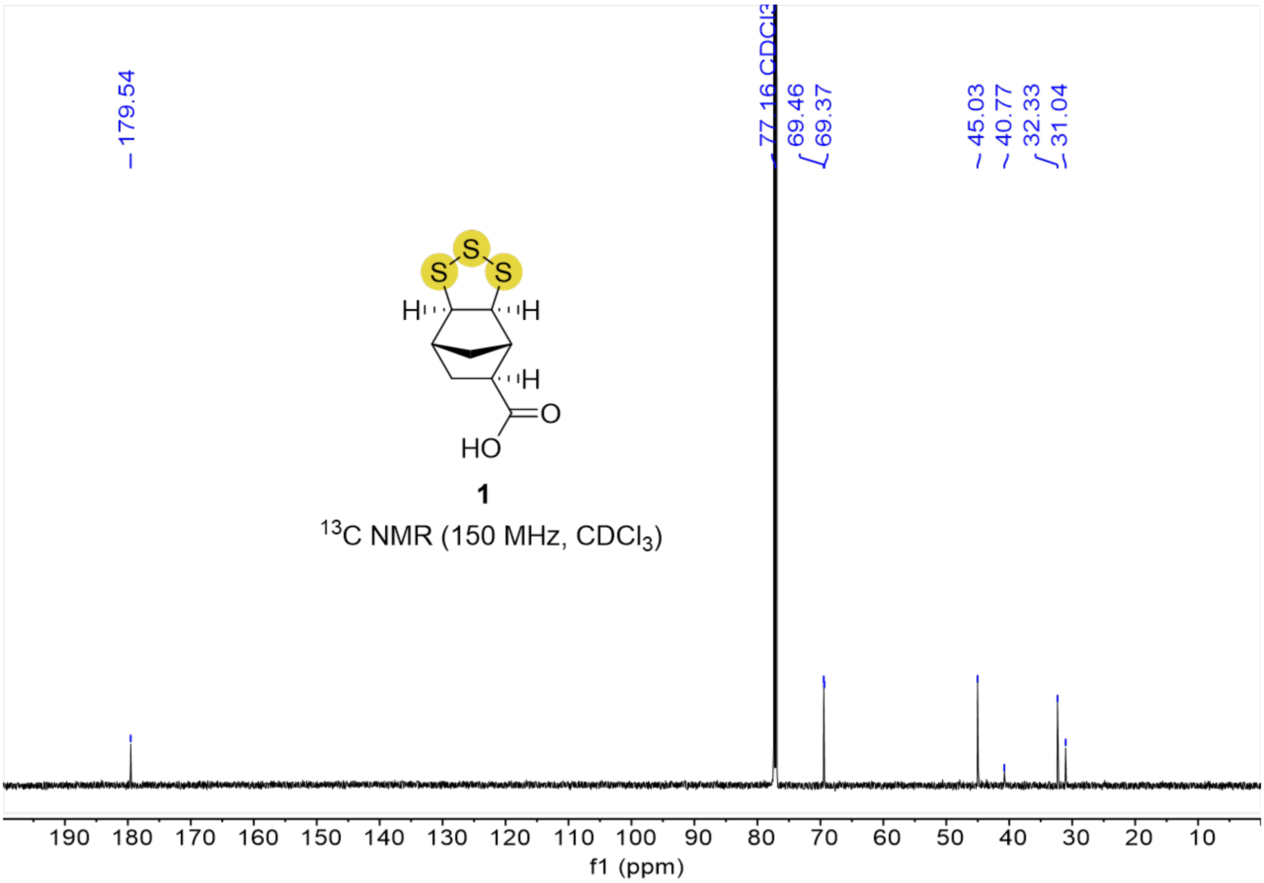

^{13}C NMR spectrum (150 MHz, $CDCl_3$) of monomer **1**. ^{13}C NMR (151 MHz, $CDCl_3$): δ 179.5, 69.3, 45.0, 40.7, 32.3, 31.0.

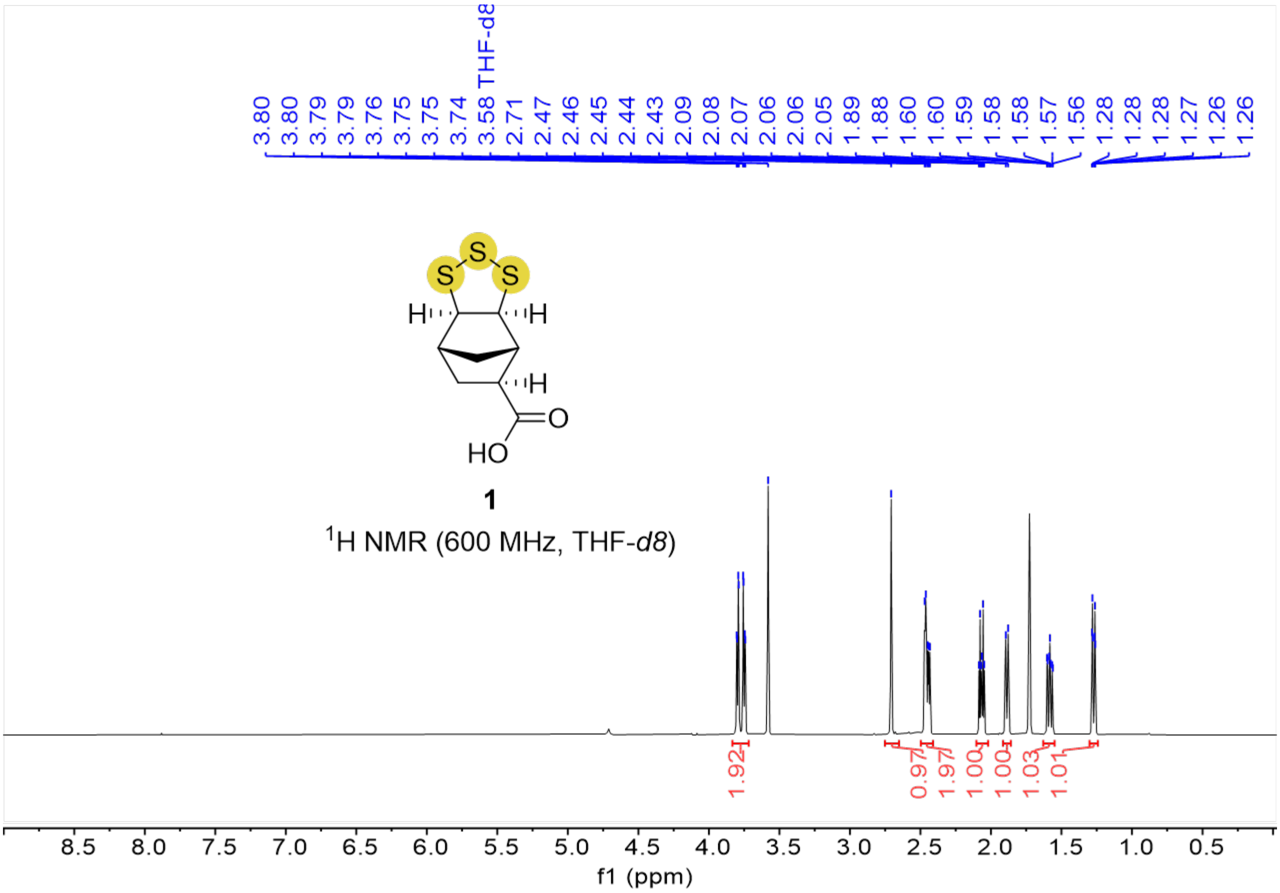

^{1}H NMR spectrum (600 MHz, THF-$d8$) of monomer **1**. ^{1}H NMR (600 MHz, THF-$d8$) δ 3.77 (m, 2H), 2.71 (ap. s, 1H), 2.51 – 2.42 (m, 2H), 2.07 (dt, J = 12.8, 4.8 Hz, 1H), 1.89 (m, 1H), 1.58 (m, 1H), 1.27 (m, 1H).

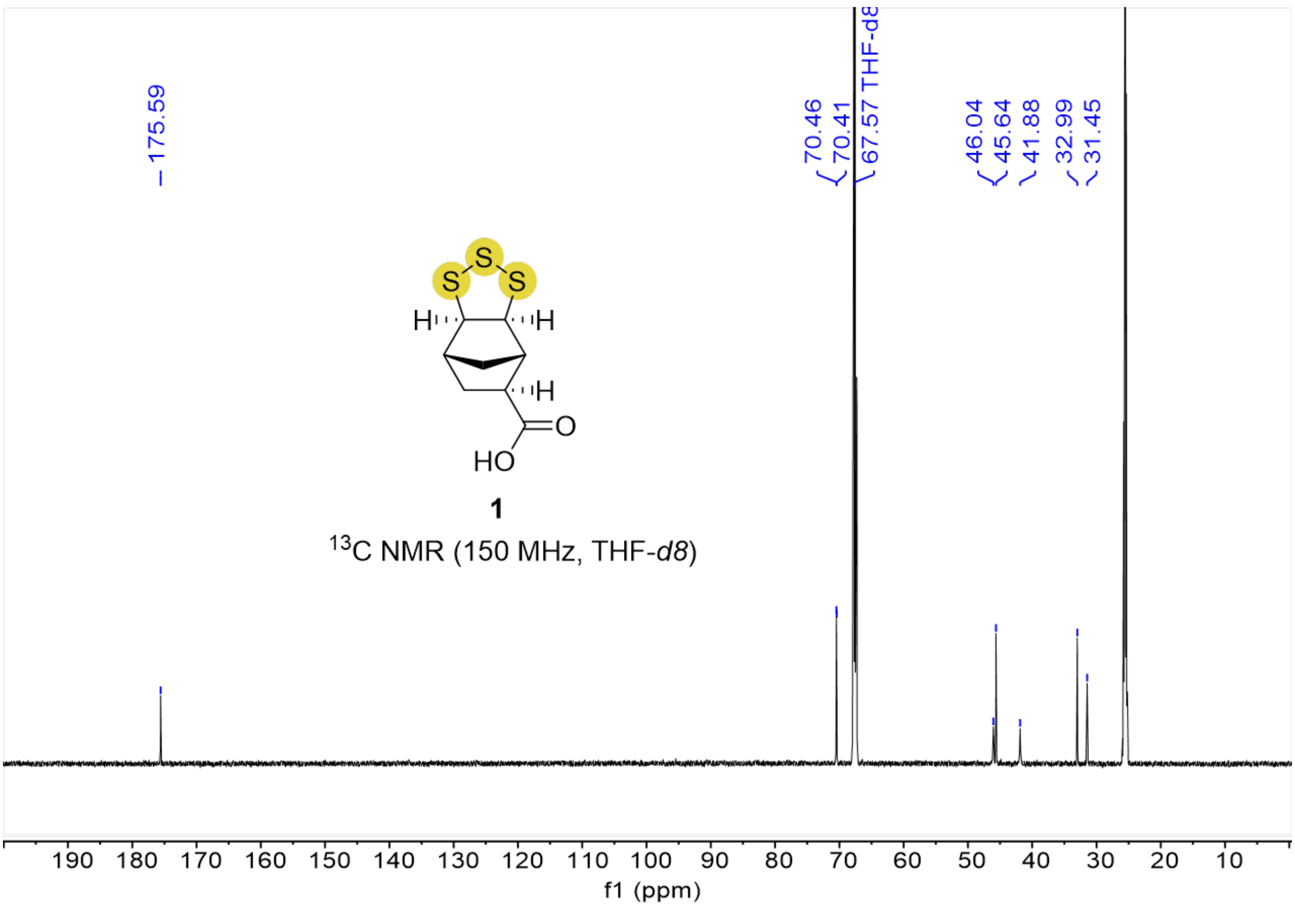

^{13}C NMR spectrum (150 MHz, THF-*d8*) of monomer **1**. ^{13}C NMR (150 MHz, THF-*d8*) δ 175.59, 70.46, 70.41, 46.04, 45.64, 41.88, 32.99, 31.45.

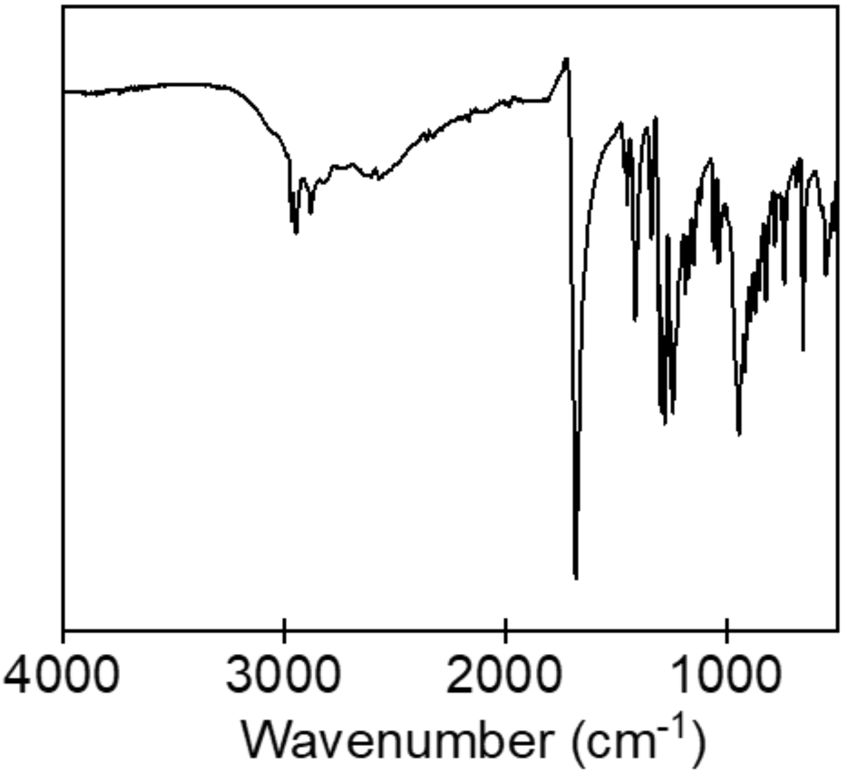

IR spectrum of monomer **1** (neat).

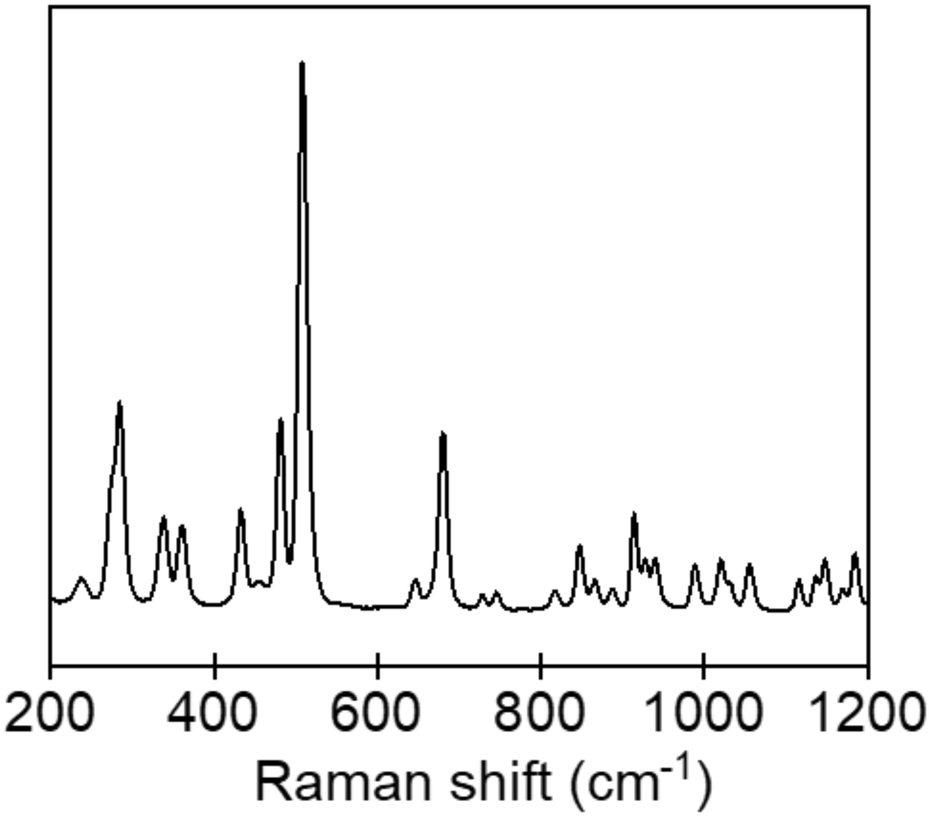

Raman spectrum of monomer **1** (neat).

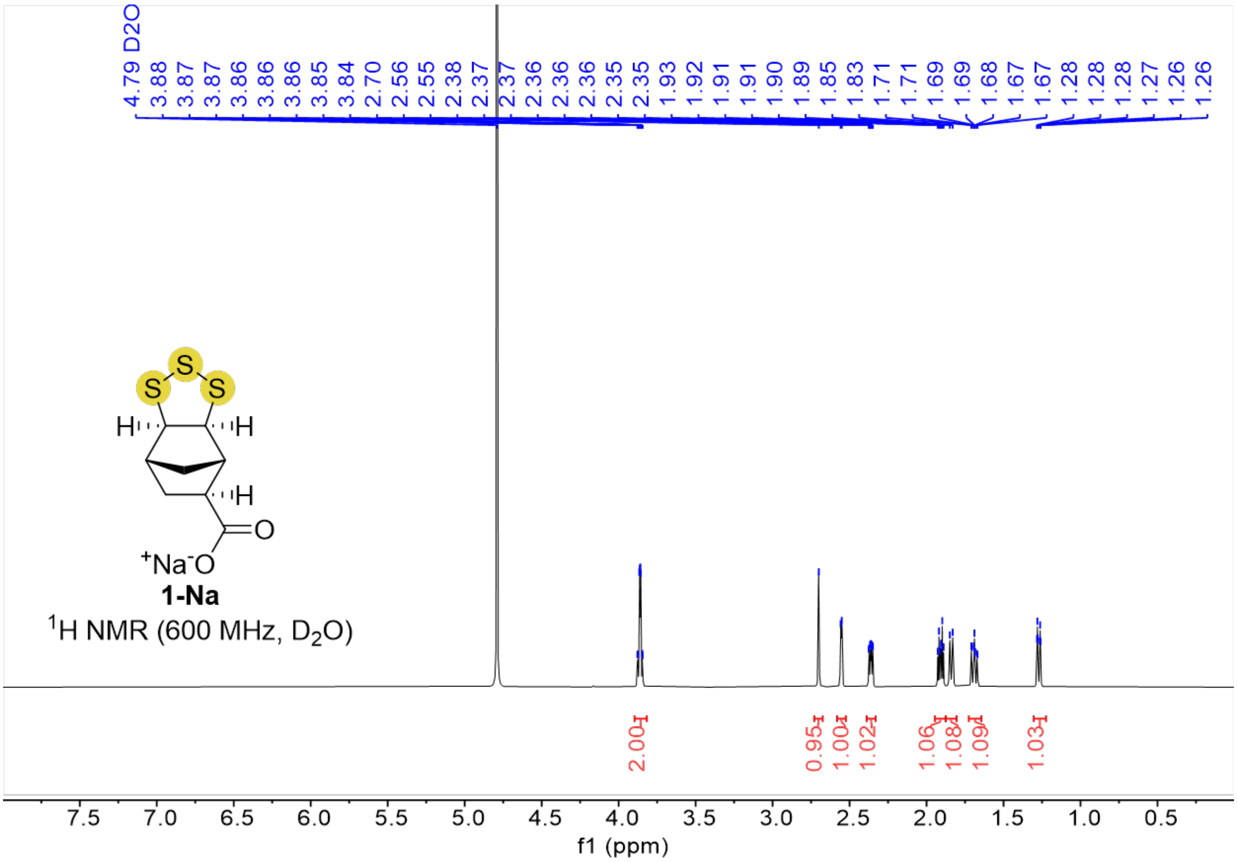

^{1}H NMR spectrum (600 MHz, D_2O) of monomer **1-Na**. ^{1}H NMR (600 MHz, D_2O) δ 3.84 – 3.88 (m, 2H), 2.70 (ap. s, 1H), 2.55 (ap. d, J = 4.5 Hz, 1H), 2.35 – 2.38 (m, 1H), 1.89 – 1.93 (m, 1H), 1.83 – 1.85 (m, 1H), 1.67 – 1.71 (m, 1H), 1.26 – 1.28 (m, 1H).

This spectrum demonstrates the water-solubility of monomer **1** after deprotonation.

Poly-1 characterization

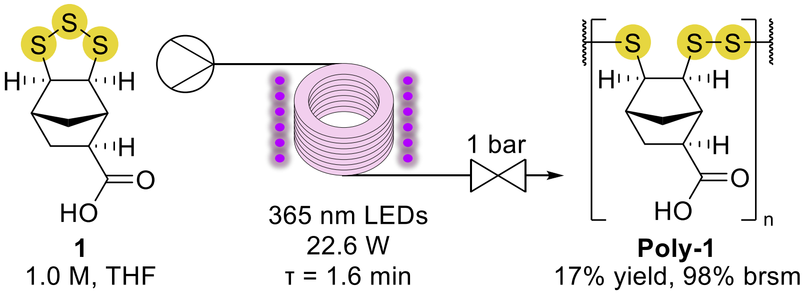

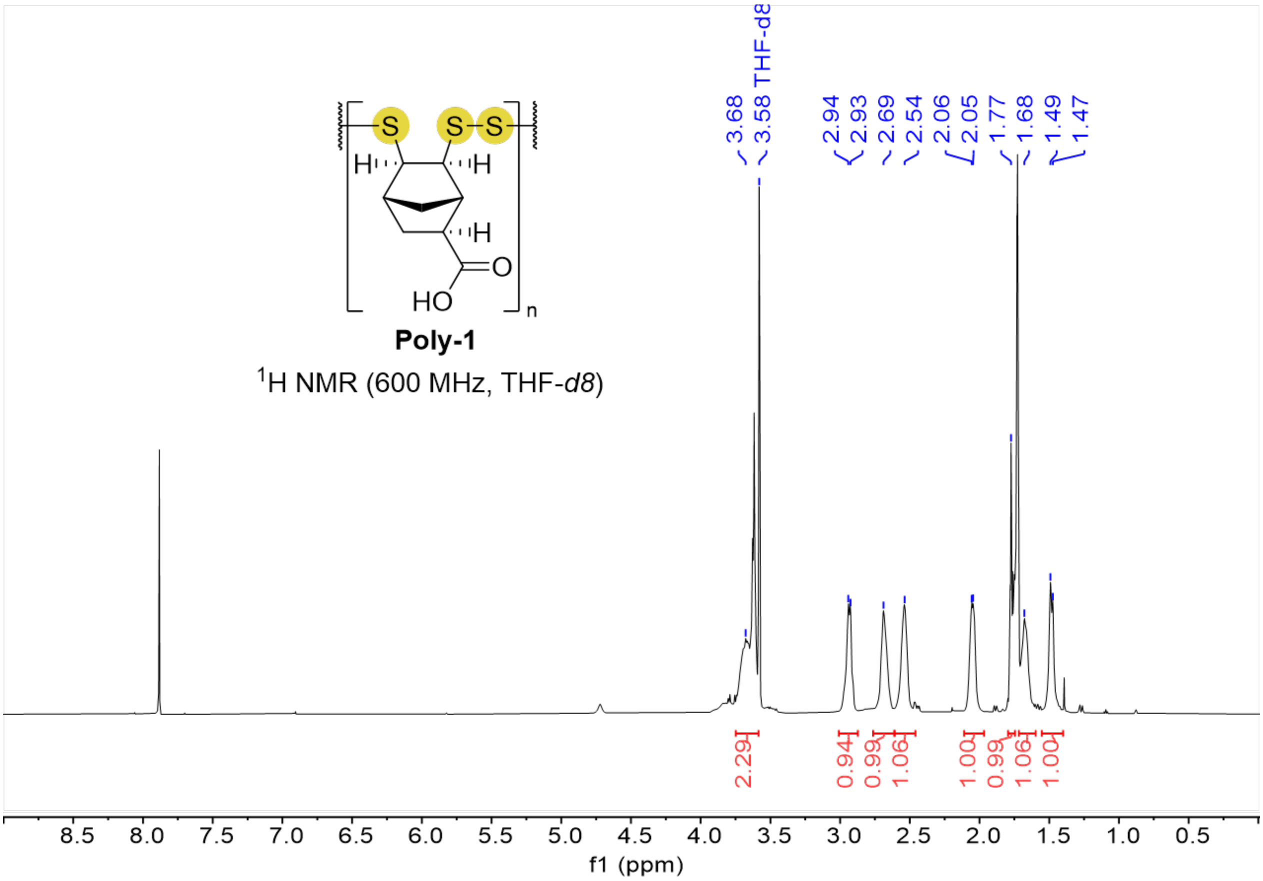

^{1}H NMR spectrum (600 MHz, THF-*d8*) of **poly-1**.

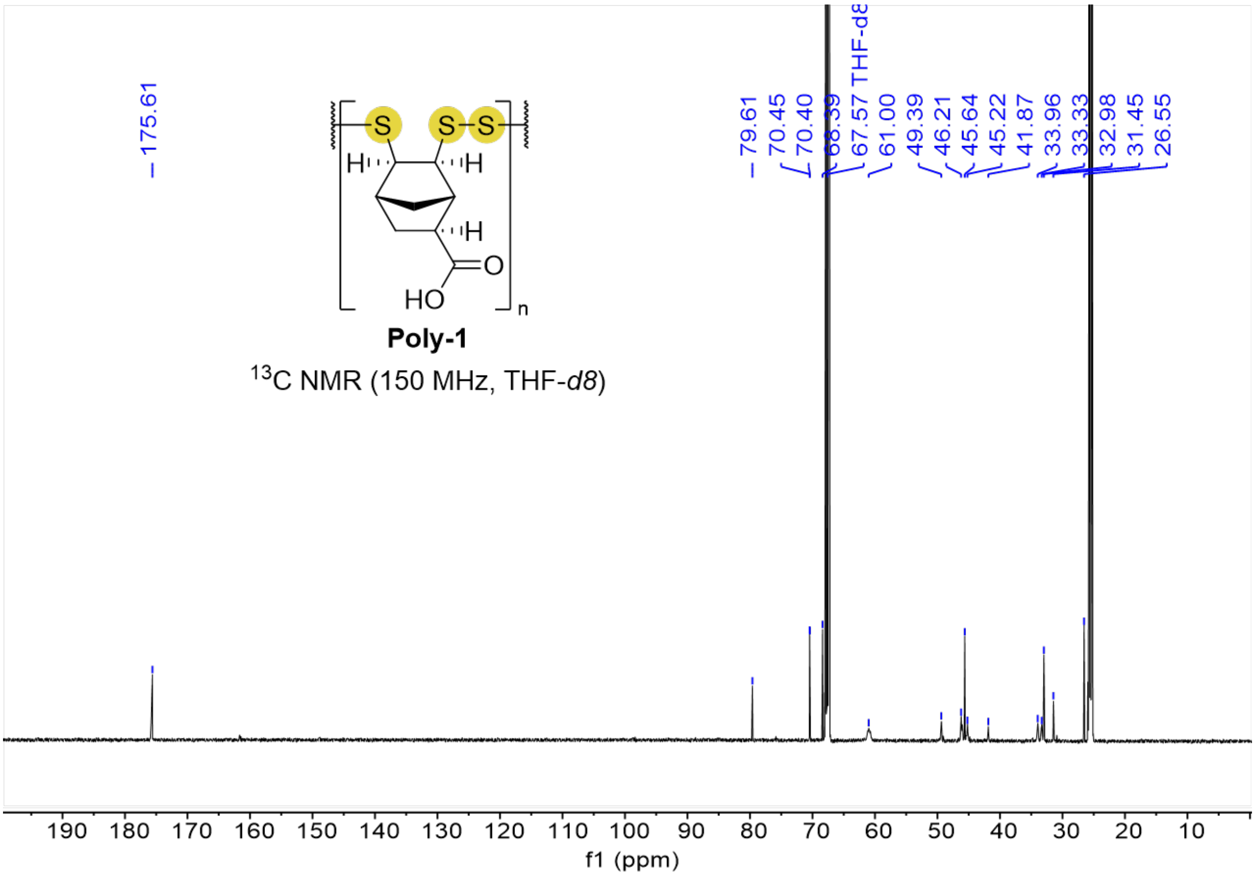

^{13}C NMR spectrum (150 MHz, THF-*d8*) of **poly-1**.

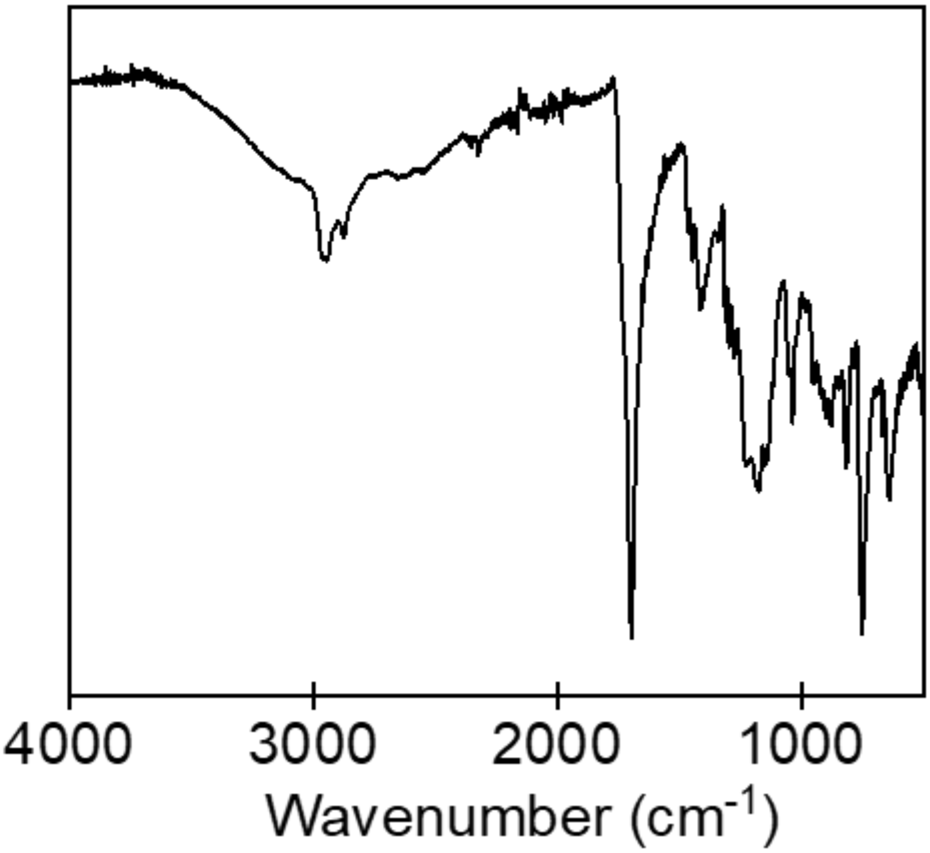

IR spectrum of **poly-1** (neat).

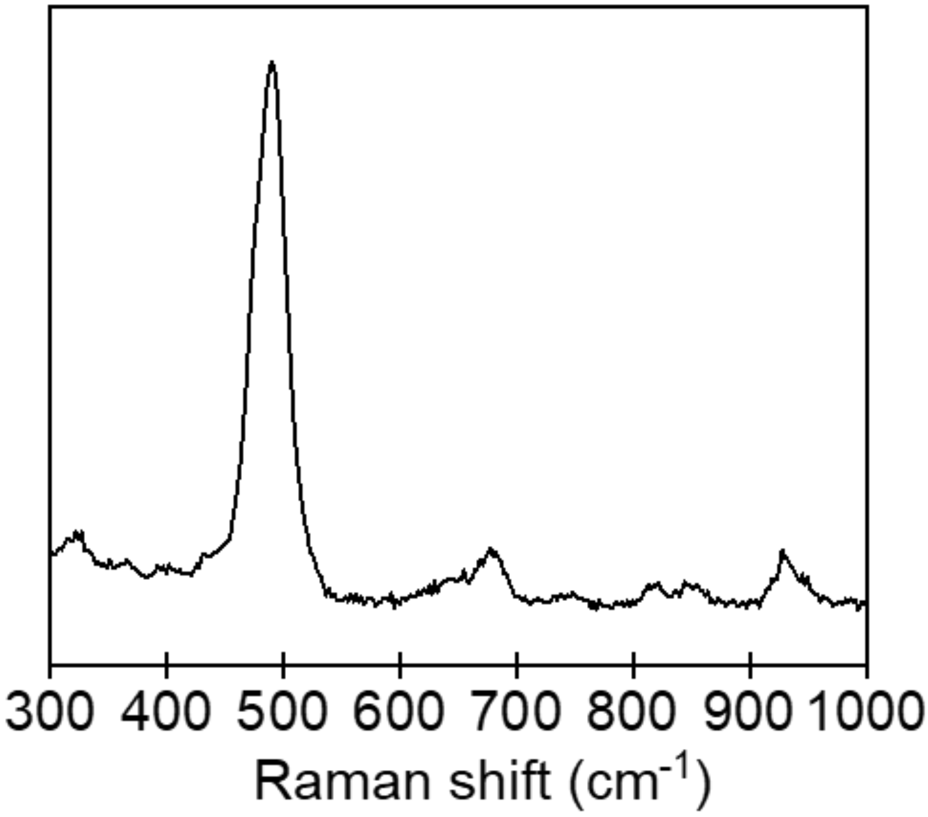

Raman spectrum of **poly-1** (neat).

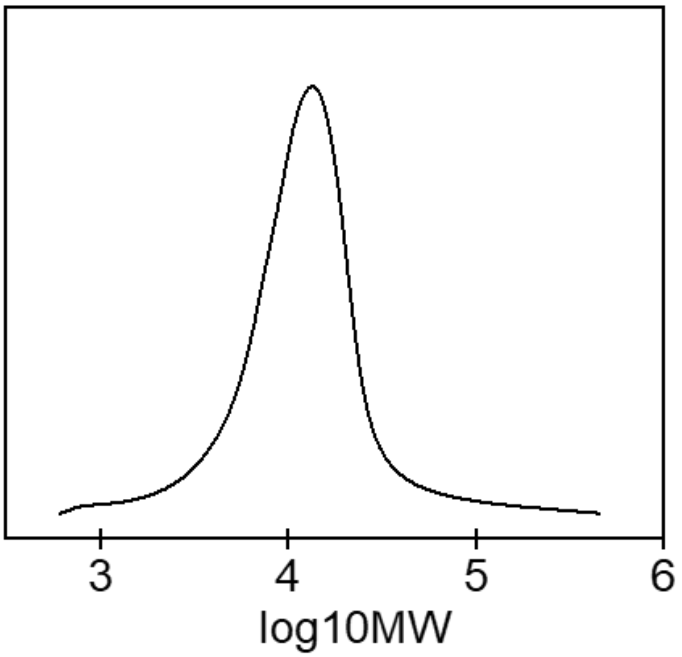

Gel permeation chromatogram (THF) of **poly-1** 16,580 g/mol with a dispersity of *Đ* = 1.89.

Batch-to-batch reproducibility of poly-1 synthesis

Poly-1 synthesis: A solution of **1** (4 mmol, 1.0 M) in THF (4 mL) was prepared. The solution was pumped through perfluoroalkoxyalkanes (PFA) tubing (10 mL, 21 °C) at a set flow rate with back pressure regulation at 1 bar. The monomer solution was irradiated continuously by light emitting diodes (LEDs) (365 nm, 22.6 W) in the flow reactor. The outflow of the reactor was collected in a round bottom flask. The solvent was removed under reduced pressure, and the resulting residue was redissolved in THF (2 mL) and then precipitated by adding chloroform (20 mL). The precipitated polymer was collected by filtration and redissolved in THF (2 mL) before precipitation into chloroform (20 mL). This process was repeated three times to purify the polymer from unreacted monomer. The resulting purified polymer was analyzed by GPC (THF). The combined filtrate was concentrated under reduced pressure and analyzed by ^{1}H NMR ($CDCl_3$) spectroscopy to recover pure monomer **1**.

Summary of GPC data of the polymerization of monomer **1** (1.0 M, THF) at varying residence times. The first three entries are triplicate runs with 1.6 min residence time and entries 4 and 5 are duplicates of runs with 2.5 min residence time.

	Flow rate (mL/ min)	Residence time (min)	M_w (g/mol)	*Đ*	**Poly-1** yield (%)
1	6	1.6	16,580	1.86	17
2	6	1.6	16,920	2.11	10
3	6	1.6	15,450	2.11	13
4	5	2.5	11,350	1.97	21
5	5	2.5	11,250	1.63	19
6	10	1.0	25,580	2.35	12

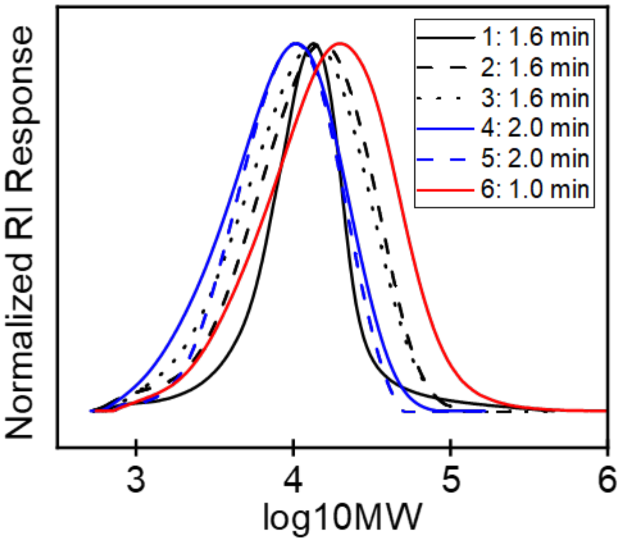

Gel permeation chromatogram (THF) of **poly-1** at varying residence time.

Quantification of apparent thiol and perthiol end groups using Ellman's Reagent

The following experiment was performed in triplicate using three flat-bottom 96-well plates. To generate a calibration curve for thiol quantification, a stock solution of L-glutathione (1.6 mM) was prepared in phosphate buffer (100 mM, pH 7.5). A 200 µL aliquot was dispensed into well A1 and 100 µL of phosphate buffer (100 mM, pH 7.5) was dispensed into wells A2-A12. A two-fold serial dilution was then performed across the row (A1-A12). Stock solutions of **1-Na** and **poly-1-Na** were prepared separately by adding 37.5 mg of either compound (0.16 mmol COOH) to a vial, followed by 840 µL of phosphate buffer (100 mM, pH 7.5). As neither compound dissolved in buffer alone, 160 µL of 1.0 M NaOH (0.16 mmol) was added to each vial. A control solution was prepared containing 840 µL of phosphate buffer (100 mM, pH 7.5) and 160 µL of 1.0 M NaOH (0.16 mmol). 200 µL of the **1-Na** solution was dispensed into well B1 and C1, 200 µL of the **poly-1-Na** solution was dispensed into well D1 and E1, and 200 µL of the NaOH control solution was dispensed into well F1 and G1. 100 µL of phosphate buffer (100 mM, pH 7.5) was added to wells B2-B12, C2-C12, D2-D12, E2-E12, F2-F12 and G2-G12. Two-fold serial dilutions were then performed across each row. Following preparation of the dilution series, 50 µL of Ellman's Reagent (10 mM in phosphate buffer, 100 mM, pH 7.5) was added to each well in row A, B, D and F. The plate was incubated at room temperature for 20 minutes. After incubation, the absorbance of each well at 412 nm was measured using a FLUOstar Omega microplate reader. The concentration of **1-Na** and **poly-1-Na** (10 mM of COOH) was selected because the corresponding NaOH control at this concentration had negligible absorbance at 412 nm, ensuring that the base did not interfere with thiol quantification.

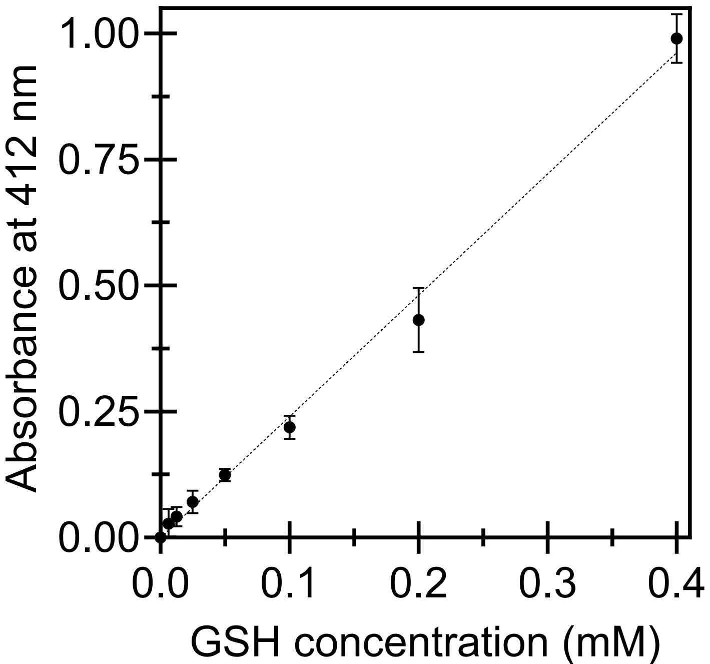

Calibration curve of absorbance at 412 nm for L-glutathione (GHS) at varying concentrations following reaction with Ellman's Reagent. Data points represent the mean of three replicates (n = 3), and error bars indicate the standard error of the mean. Linear regression was performed in GraphPad Prism, and the resulting calibration equation was used to determine the concentration of apparent thiol or perthiol species in **1-Na** (10 mM) and **poly-1-Na** (10 mM relative to the carboxylate group), as well as the NaOH control (10 mM).

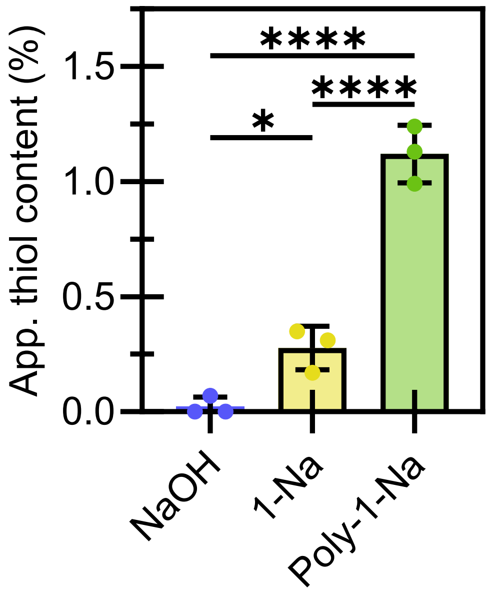

Calculated apparent thiol content in NaOH (10 mM), Na-1 (10 mM), and poly-1-Na (10 mM relative to the carboxylate group). Data represents the mean of three replicates (n = 3) and error bars show standard error of the mean. *$p \leq 0.05$, **$p \leq 0.01$, ***$p \leq 0.001$, ****$p \leq 0.0001$.

Depolymerization of poly-1 using excess NaOH

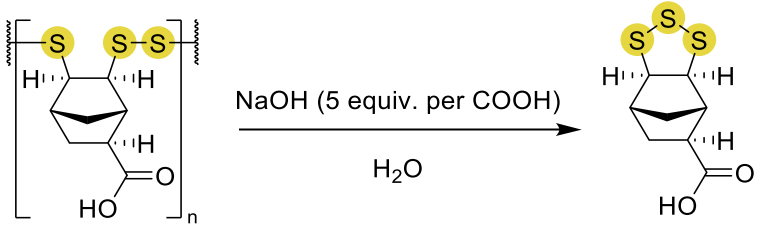

Poly-1 M_W = 11,250 g/mol, $Đ$ = 1.6

1 61% yield

A 20 mL glass vial was charged with **poly-1** (99.8 mg, M_W = 11,250 g/mol, $Đ$ = 1.6), followed by the addition of deionized water (1.0 mL), and then a solution of NaOH (2.31 mL, 1.0 M in H_2O; 5 equivalents per carboxylic acid group). The resulting mixture was vortexed for 1 minute and immediately transferred to a separating funnel. HCl (10 mL, 3.0 M in H_2O) was added to reprotonate the carboxylate groups, followed by extraction into chloroform (10 mL). The aqueous layer was subsequently extracted twice with chloroform (2 x 10 ml). The combined organic extracts were dried over $MgSO_4$, filtered, and concentrated under reduced pressure. A crystalline product was observed in the resulting residue (79 mg) and was further purified by passing through a short silica plug (1:9, ethyl acetate: hexane) to yield monomer **1** (61 mg, 61% recovery) as confirmed by ^1H NMR spectroscopy (see below). The melting point and HRMS were also consistent with monomer regeneration

*Characterization data for **1** recovered from depolymerization of poly-**1** during reaction with excess NaOH*

^{1}H NMR (600 MHz, $CDCl_3$): δ 3.74 – 3.69 (m, 2H), 2.81 (ap. s, 1H), 2.56 – 2.54 (m, 1H), 2.52 – 2.47 (m, 1H), 2.15 – 2.10 (m, 1H), 2.03 – 1.98 (m, 1H), 1.68 – 1.62 (m, 1H), 1.36 – 1.32 (m, 1H).

Melting point: 141 – 143 °C

HRMS (ESI^-): $C_8H_9O_2S_3$ $[M-H]^-$, Calculated: 232.9764, Found 232.9763.

The remaining aqueous layer was filtered to collect remaining solids, which were washed with deionized water (5 x 10 mL), dissolved in THF, dried over $MgSO_4$, filtered, and concentrated under reduced pressure. The resulting material was analysed by GPC (28 mg, M_W = 1,260 g/mol, $Đ$ = 1.1), indicating the presence of low molecular weight **poly-1**.

Depolymerization of poly-1 with excess NaOH (con't)

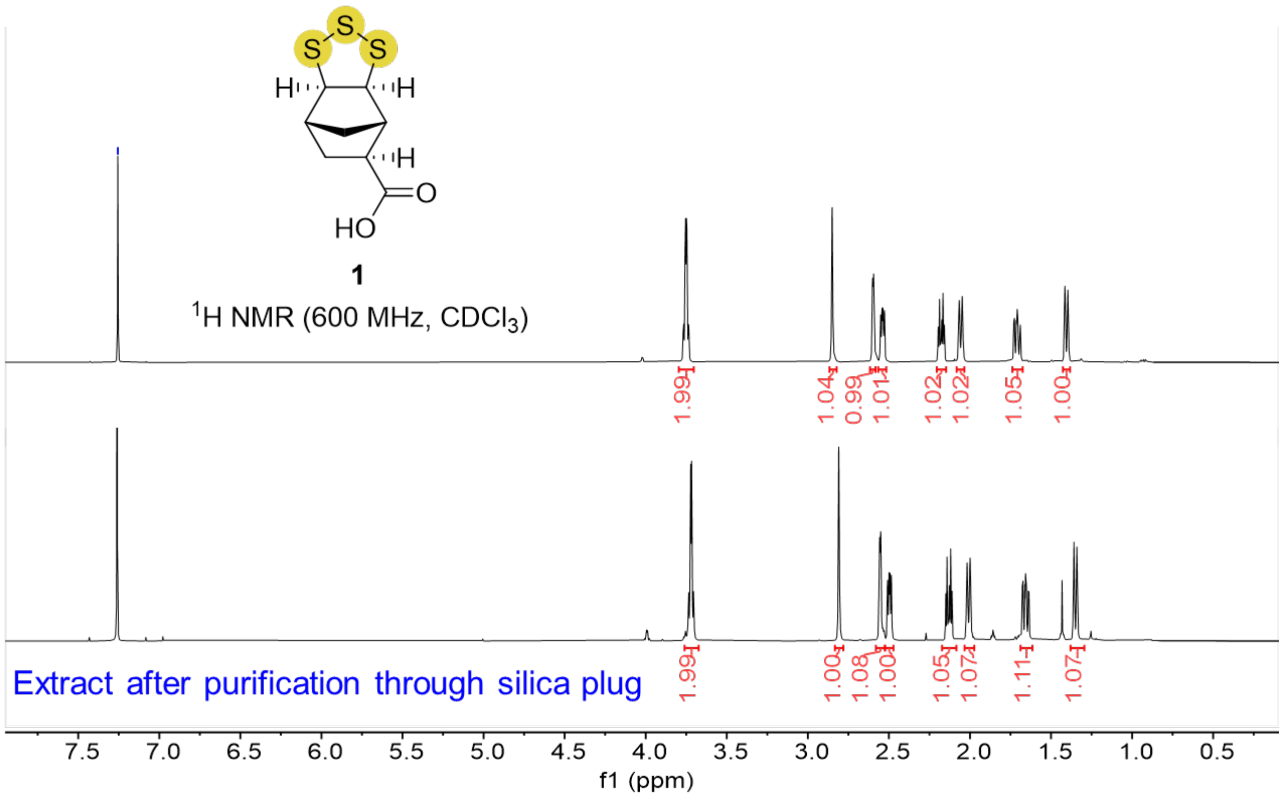

^{1}H NMR spectra (600 MHz, $CDCl_3$) of monomer **1** originally synthesised (top) and recovered monomer **1** after depolymerization using excess NaOH and purification through a short silica plug (1:9, ethyl acetate: hexane) (bottom).

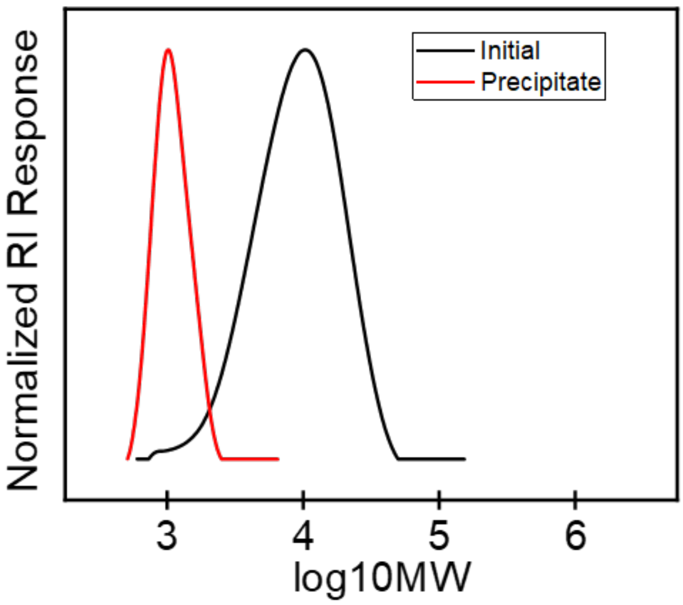

Gel permeation chromatograms (THF) of **poly-1** (M_W= 11,250 g/mol, $Đ$ = 1.6, black) and recovered oligomeric material (M_W = 1,260 g/mol, $Đ$ = 1.1, red) after depolymerization with excess NaOH.

Reaction of two poly-1 samples of different M_W with NaOH

.

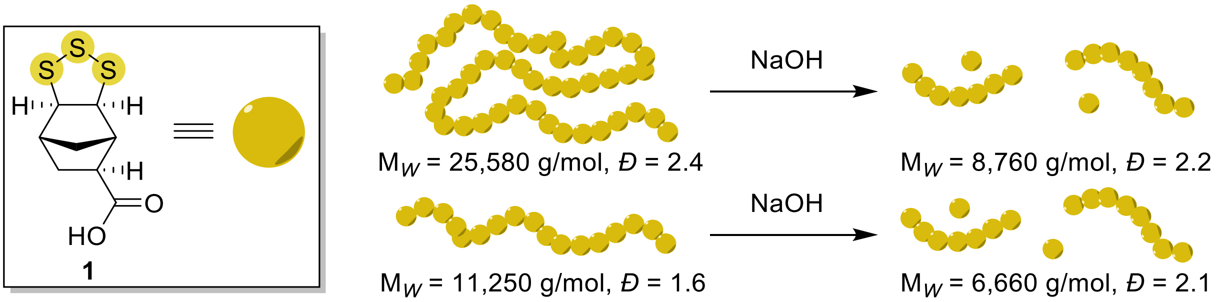

Two samples of **poly-1** were prepared with the following molecular weights:

(i) Low molecular weight sample: M_W = 11,250 g/mol, Đ = 1.6

(ii) High molecular weight sample: M_W = 25,580 g/mol, Đ = 2.4

Each sample (20 mg, 0.085 mmol carboxylic acid groups) was suspended in deionized water in a separate vial, followed by the addition of NaOH (85 µL, 1.0 M in H_2O, 0.085 mmol). This amount of NaOH corresponds to 1 molar equivalent, relative to the carboxylic acid groups in each polymer. The mixtures were vortexed for 1 minute, resulting in dissolution of the polymer and chain scission. HCl (28 µL, 3.0 M in H_2O) was added causing the products to precipitate from the solution. The precipitate was collected by filtration, washed with deionized water (5 x 10 mL), redissolved in THF, dried over $MgSO_4$, filtered, and concentrated under reduced pressure. Mass recovery was 19.5 mg for the low molecular weight sample and 16.5 mg for the high molecular weight sample. The samples were then dissolved in THF and analysed by GPC. These results indicate that the chain scission provides a similar molecular weight and distribution, independent of the molecular weight of the original polymer.

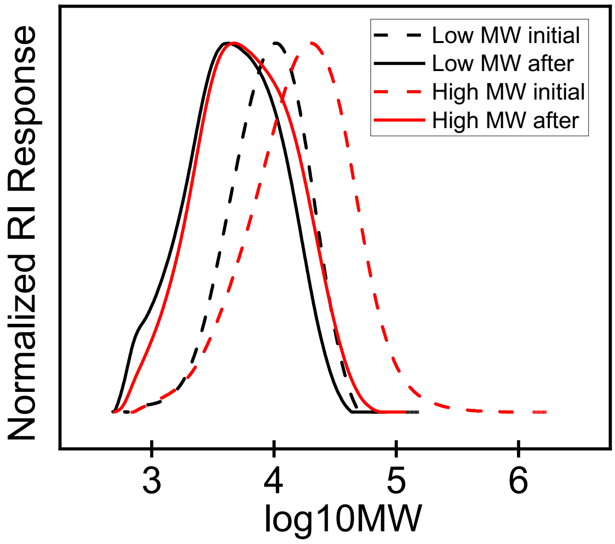

Gel permeation chromatogram (THF) of **poly-1** at high and low molecular weight (dotted lines) and the products resulting after treatment with NaOH (solid lines).

Depolymerization of poly-1 by reaction with DMSO

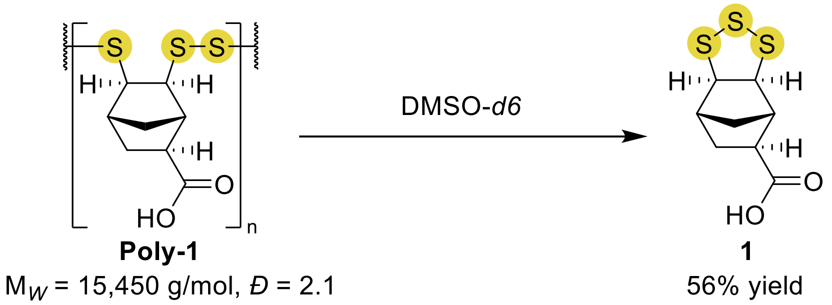

Poly-1 (68 mg, M_W = 15,450 g/mol, Đ = 2.1) was dissolved in DMSO-*d6* by vortexing for 1 minute, resulting in complete dissolution. The resulting solution was analyzed by ^{1}H NMR spectroscopy. The solution was then transferred to a separating funnel, diluted with chloroform (20 mL), and washed with deionized water (5 x 10 mL) to remove DMSO. The organic phase was dried over $MgSO_4$, filtered, and concentrated under reduced pressure. The residue was passed through a short silica plug (1:9, ethyl acetate: hexane) to yield monomer **1** (39 mg, 56% yield) as confirmed by ^{1}H NMR spectroscopy, melting point, and HRMS.

^{1}H NMR (600 MHz, $CDCl_3$): δ 3.74 – 3.69 (m, 2H), 2.81 (ap. s, 1H), 2.56 – 2.54 (m, 1H), 2.52 – 2.47 (m, 1H), 2.15 – 2.10 (m, 1H), 2.03 – 1.98 (m, 1H), 1.68 – 1.62 (m, 1H), 1.36 – 1.32 (m, 1H).

Melting point: 141 – 143 °C

HRMS (ESI^-): $C_8H_9O_2S_3$ $[M-H]^-$, Calculated: 232.9764, Found 232.9762.

The aqueous phase was filtered to recover a precipitated solid which was washed with H_2O (5 x 10 mL), dissolved in THF, dried over $MgSO_4$, filtered, and concentrated under reduced pressure to afford 6 mg of material. This extract was analyzed by GPC (THF).

Depolymerization of poly-1 by reaction with DMSO (continued)

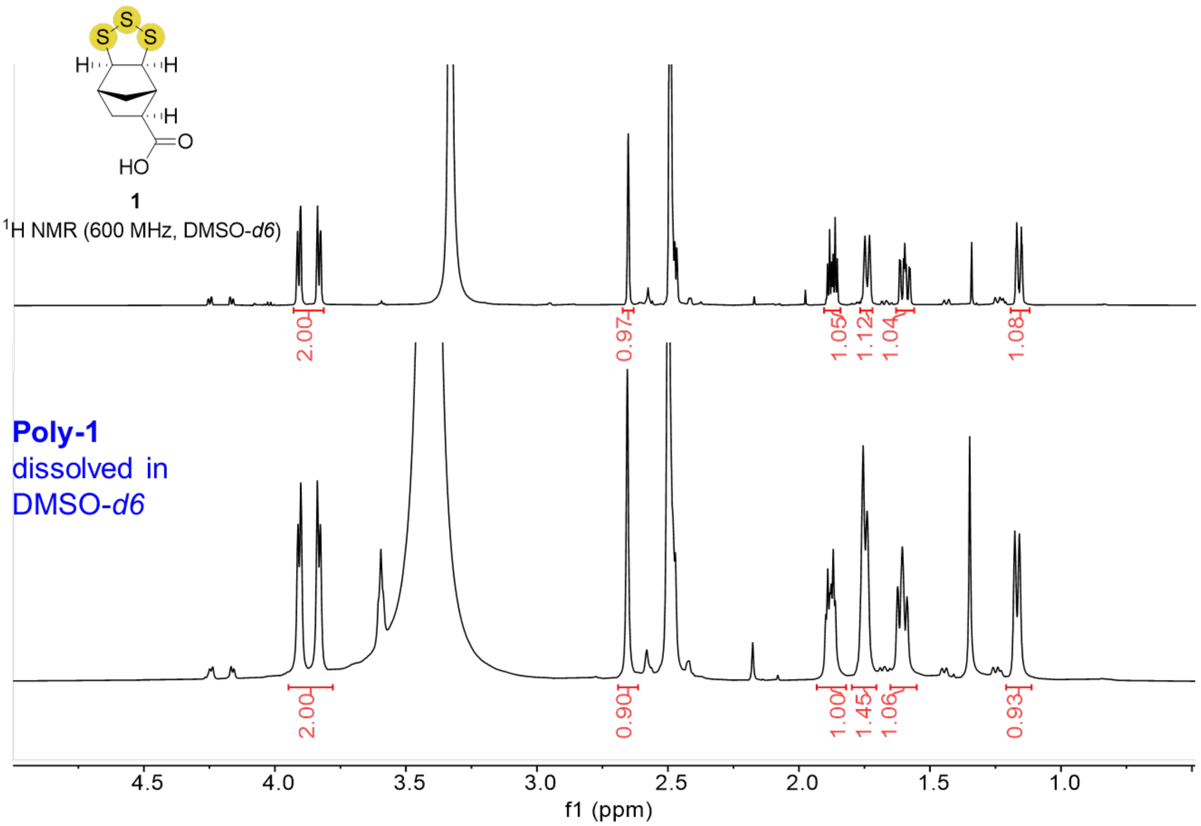

^{1}H NMR spectra (600 MHz, DMSO-d6) of monomer **1** (top) and **poly-1** dissolved in DMSO-*d6* (bottom). The monomer was regenerated upon reaction of DMSO with **poly-1**.

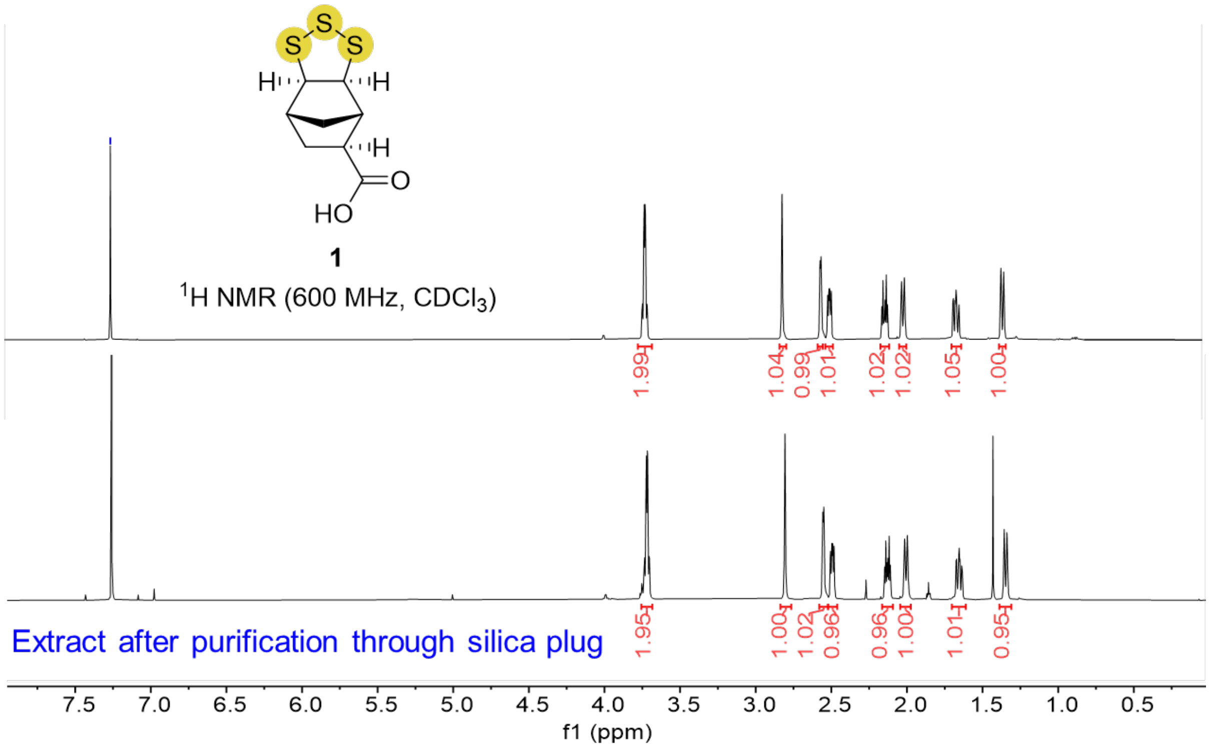

^{1}H NMR spectra (600 MHz, $CDCl_3$) of monomer **1** as originally synthesized (top) and recovered monomer **1** after depolymerization during the reaction of DMSO and **poly-1**.

The regeneration monomer was purified by passing through a short silica plug (1:9, ethyl acetate: hexane) (bottom).

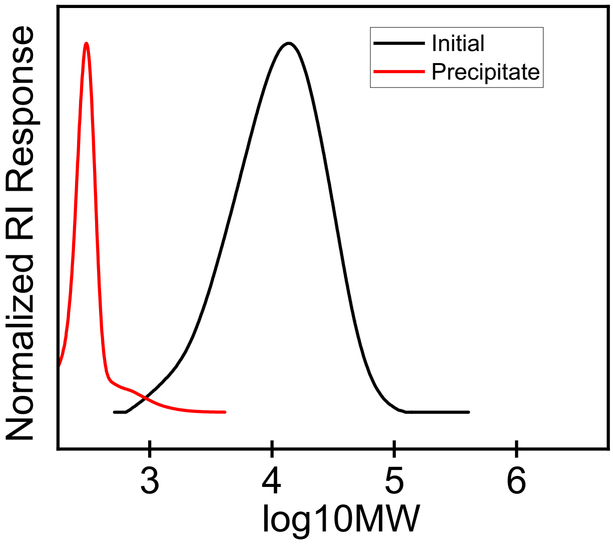

Gel permeation chromatogram (THF) of the initial **poly-1** (M_W = 15,450 g/mol, $Đ$ = 2.1) and the oligomer isolated (9% of original polymer mass) after the depolymerization.

Quantitative PLSR analysis by Raman spectroscopy

A series of seven mixtures were prepared containing **poly-1** and monomer **1.** The details of the sample mixtures are given in the table below. These samples were used to quantify the amount of polymer in the sample, **poly-1***.

Table S1: Reference mixture dataset based on the calculated % mass composition.

**Sample**	%w/w **Poly-1**	%w/w **1**
0	0.0	100.0
25	25.0	75.0
33	33.3	66.6
50	50.0	50.0
66	66.6	33.3
75	75.0	25.0
100	100.0	100.0

Data analysis: The spectral data were analysed using The Unscrambler X V.11.0 (Camo Analytics, Oslo, Norway). Spectra underwent a standard normal variate scatter and scale correction over the spectral region 380 to 600 cm^{-1}. These pre-processed spectra were then used to create a partial least squares regression (PLSR) model to quantify the relative abundance of monomer to polymer present. The PLSR model was created with the Kernal PLS method using the model set data (0, 25, 50, 78 and 100 % polymer spectra) with cross validation (random, 3 samples per segment) to identify the appropriate number of factors to use. The model was applied to the test set spectra (0, 33, 66 and 100 % polymer) to evaluate the model performance prior to being applied to the unknown sample.

Results

The spectral series visually follow the loss of the band at 512 cm^{-1} and growth of the band at 490 cm^{-1} with increased polymer content (Figure S1). There were also notable differences between replicate spectra of the monomer, these differences are tentatively attributed to orientation effects whilst measuring the crystalline monomer.

Two factors were required for this PLSR model, whilst the first factor primarily attributed to the difference between the monomer and polymer, the second factor appears to account for the variance observed within the monomer. The 2 factor model appears sensical with the regression coefficients consistent with the differences in the features present in the monomer (negative coefficients) and polymer (positive coefficients) as seen in Figure S2. Application of the test set to the model gives a reasonable performance with a slope close to 1 (0.96) an r-squared of 0.95 and a root mean squared error of prediction (RMSEP) of 5.8 % (Figure S3). Part of this error may be associated with the small sample volumes measured with this setup (~1.3 µm Ø) which can cause sub-sampling therefore future work of similar systems should ideally explore the use of Raman systems with larger sample volumes if available. Replicate spectra gave predicted % polymer values within the range of 74.7 to 78.1 and an average of 76.8 % (Table S2). When accounting for the associated error of the model itself, the predicted % polymer for **poly-1*** sample is 77 ± 6 %.

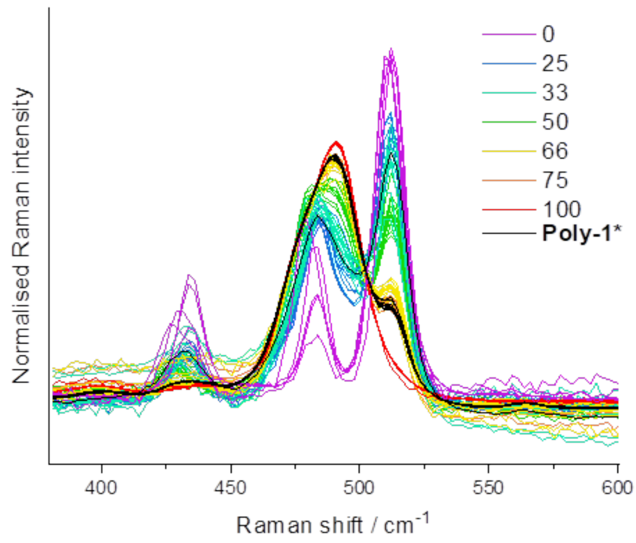

Figure S1: Representative Raman spectra from **Poly-1*** in comparison to the series of monomer to polymer mixtures (Table S1).

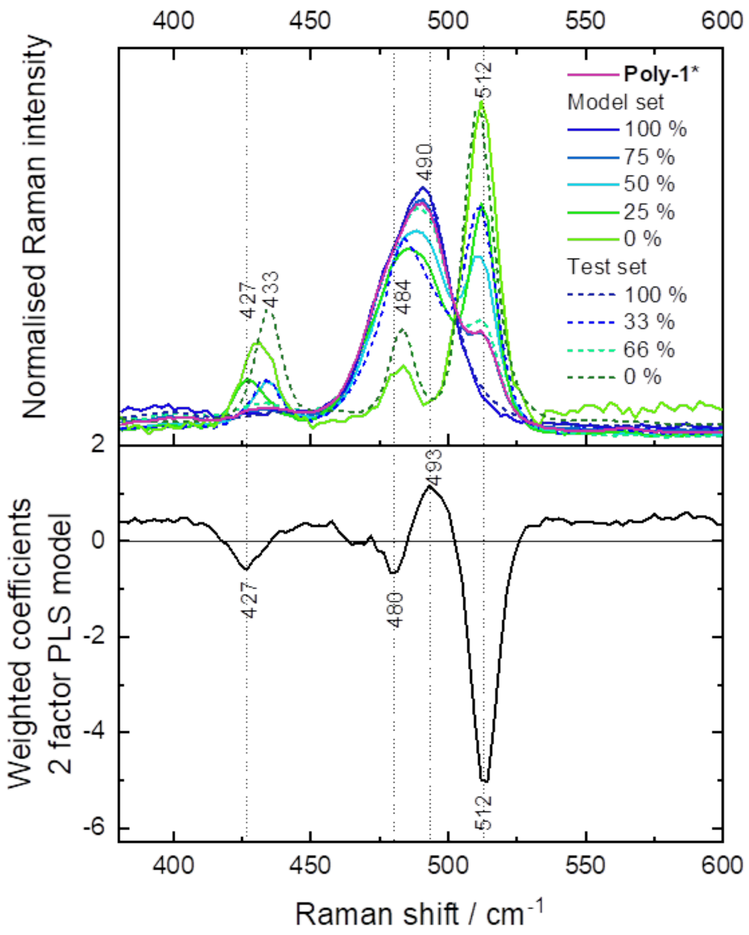

Figure S2: Representative spectra and the weighted regression coefficient for the PLSR model using 2 factors to quantify proportion of polymer.

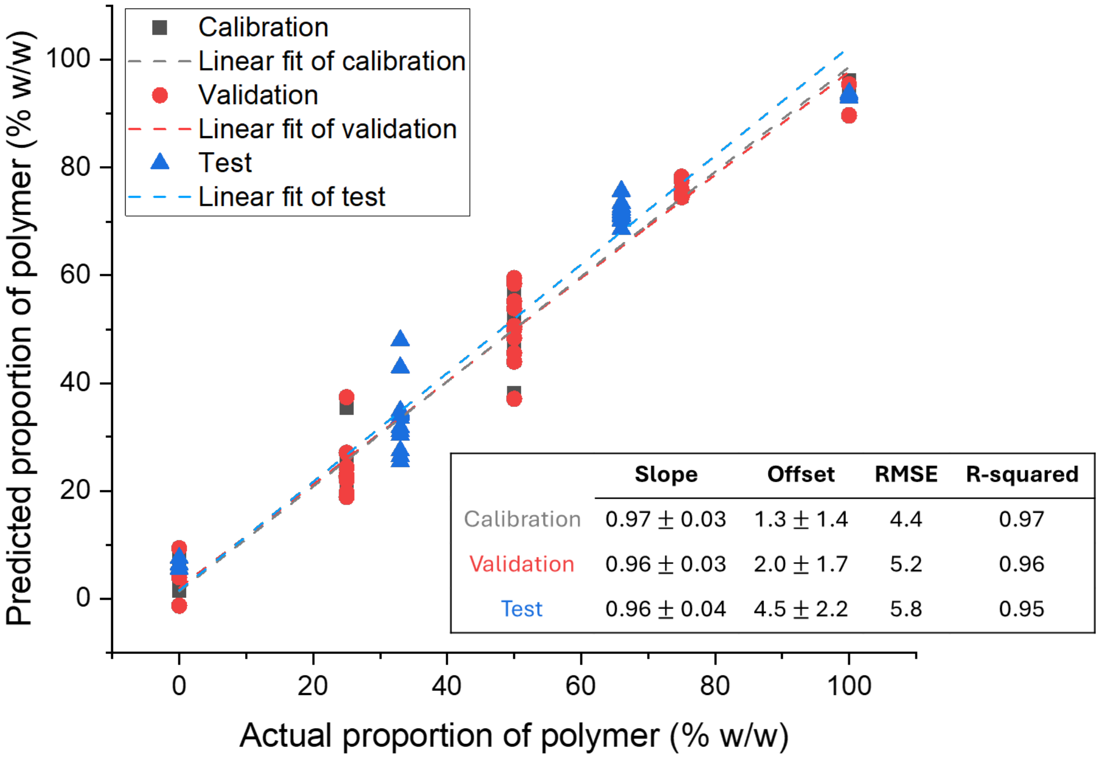

Figure S3: Predicted versus reference plot for the PLSR model performance for quantifying polymer and monomer content.

Table S2: Descriptive statistics calculated in Excel for the predicted unknown spectra (**Poly-1***) Data

Predicted % polymer
77.4
74.7
77.2
74.8
78.1
78.0
77.5
76.7

Descriptive statistics	
Mean	76.82
Standard Error	0.48
Median	77.33
Mode	#N/A
Standard Deviation	1.35
Sample Variance	1.83
Kurtosis	-0.60
Skewness	-1.00
Range	3.36
Minimum	74.74
Maximum	78.11
Sum	614.54
Count	8.00
Confidence Level (95.0%)	1.13

Summary of reaction of poly-1 with NaOH (1 equivalent of NaOH per carboxylic acid).

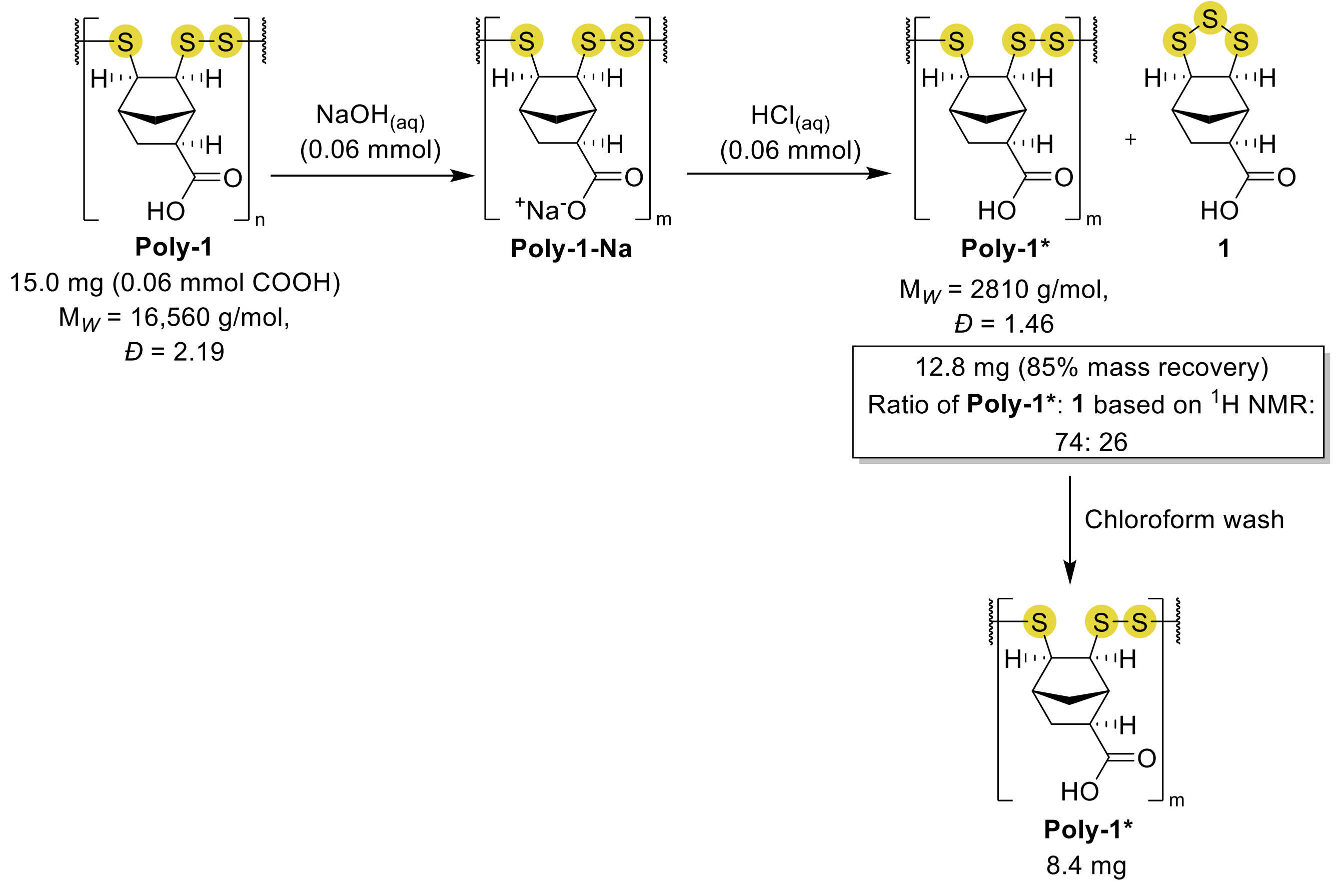

GPC analysis of poly-1 and poly-1* after removing regenerated monomer:

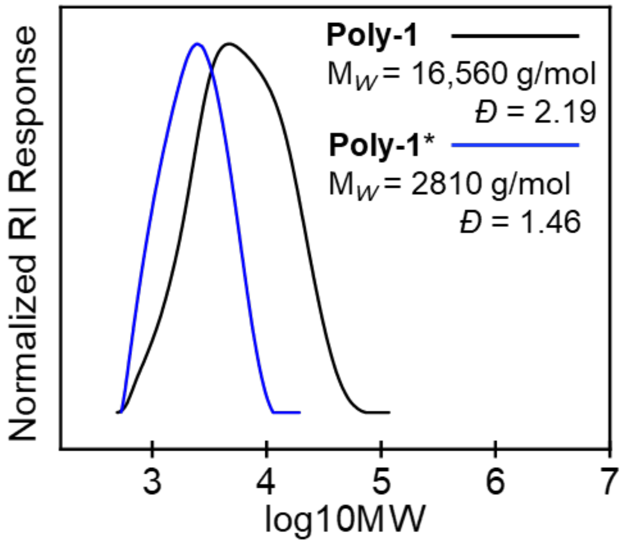

Gel permeation chromatograms (THF) of **poly-1** (M_W = 16,560 g/mol, $Đ$ = 2.19, black) and **poly-1*** (M_W = 2,810 g/mol, $Đ$ = 1.46, blue).

Summary of reaction of poly-1 with NaOH (1 equivalent of NaOH per carboxylic acid). (continued)

Raman Spectroscopy: evidence that **poly-1** and **poly-1*** both are poly(trisulfides), but differ in molecular weight. The **poly-1*** (washed) is the purified sample after washing out regenerated monomer. The peak at 489 cm^{-1} corresponds to a linear trisulfide signal.

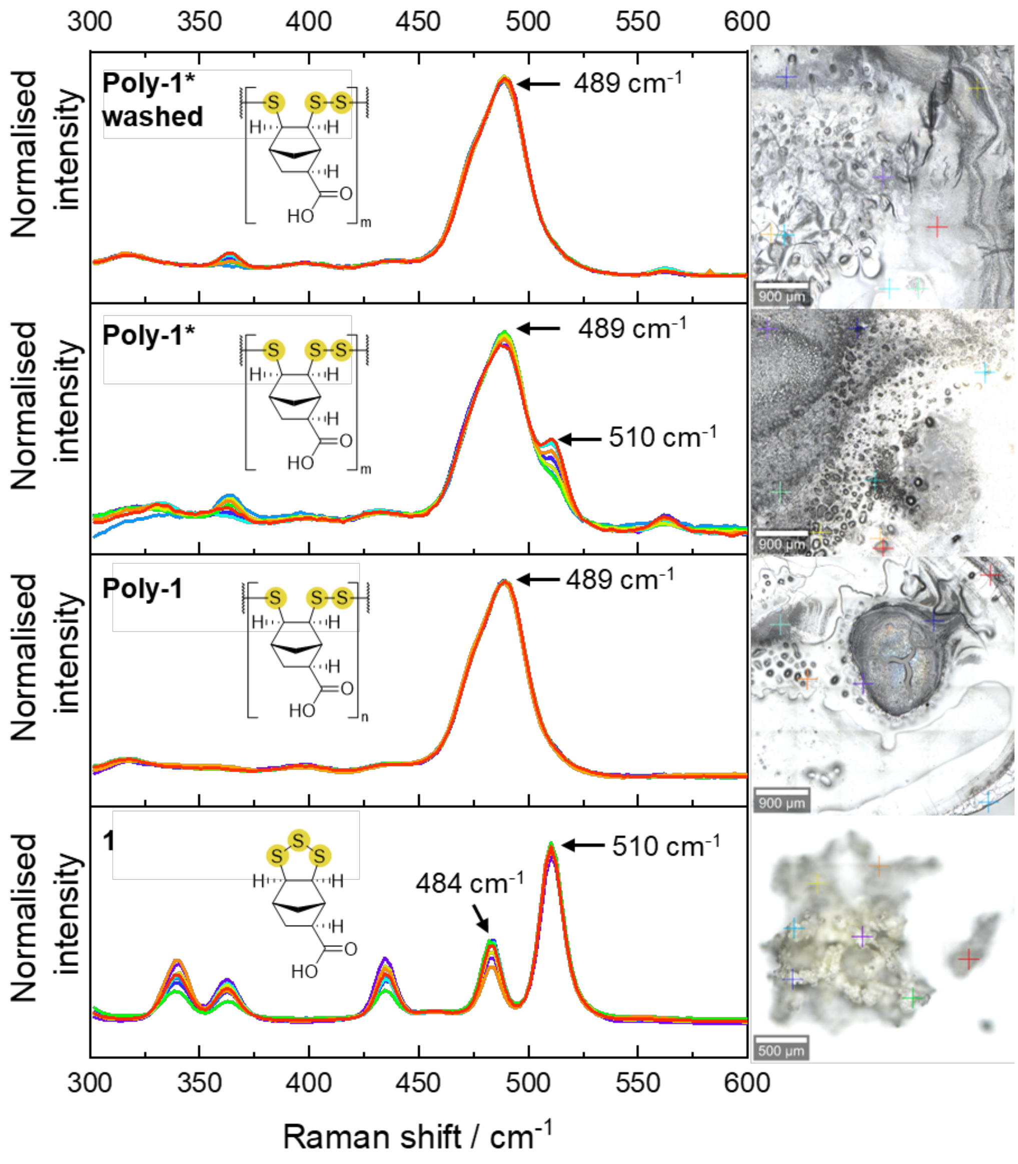

Overlay of 6 Raman spectra of **1**, **poly-1**, **poly-1***, and **poly-1*** after being washed with chloroform (3 x 10 mL) to remove regenerated **1** (left). Image of samples analyzed by Raman spectroscopy (right). The colored + on the images represents the location that the sample was analyzed. **Poly-1**, **poly-1***, and **poly-1*** were dissolved in a small amount of THF and drop-cased onto a glass slide for analysis. **1** was analyzed as a crystalline powder.

The shoulder at 510 cm^{-1} in **poly-1*** is consistent with the Raman shift observed for **1**, indicating the presence of regenerated monomer. By washing **poly-1*** with chloroform (3 x 10 mL), this peak was removed indicating the removal of **1** from **poly-1***.

Summary of reaction of poly-1 with NaOH (1 equivalent of NaOH per carboxylic acid). (continued)

^{1}H NMR Spectroscopy

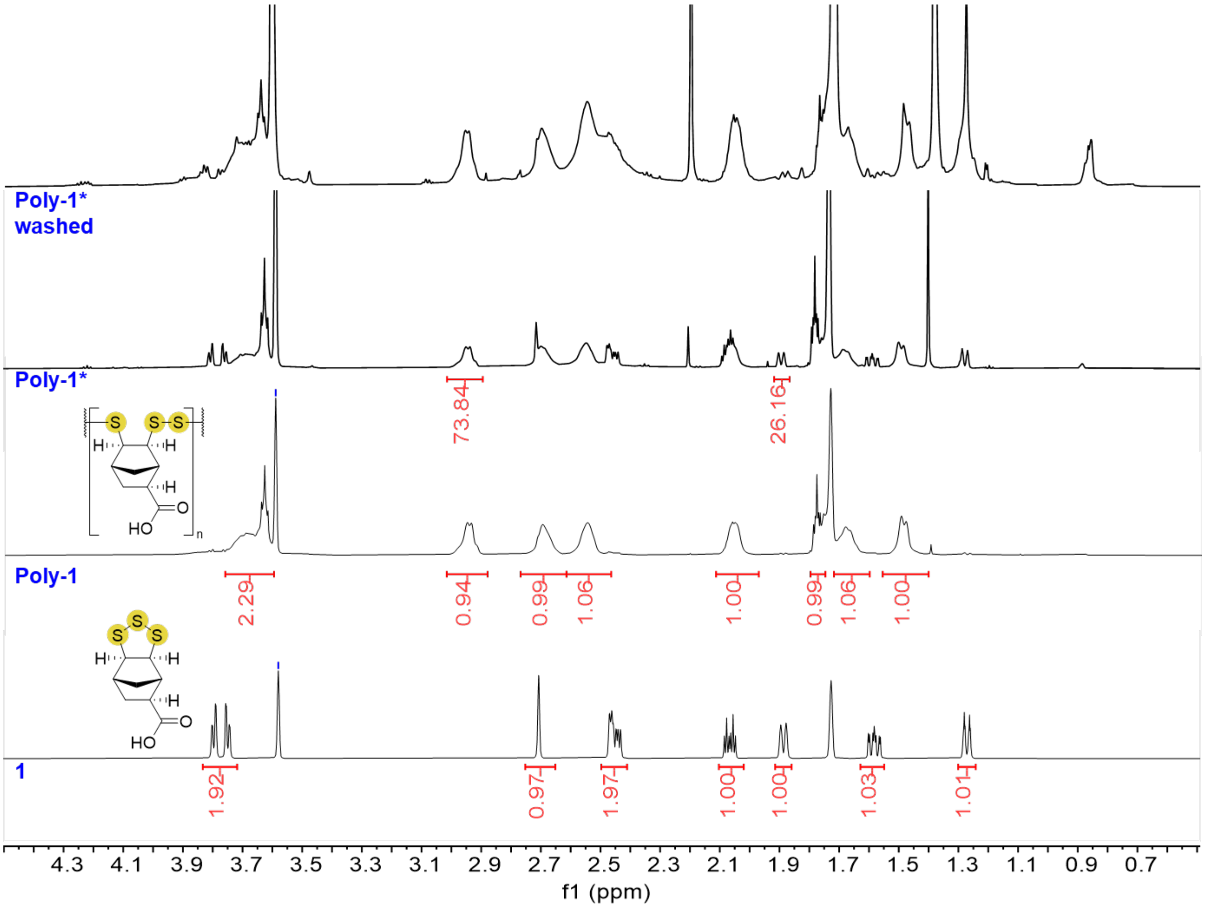

^{1}H NMR spectra (600 MHz, THF-*d8*) of **1**, **poly-1**, **poly-1***, and **poly-1*** after being washed with chloroform (3 x 10 mL) to remove regenerated **1.**

Synergy assays

Synergy assays were performed using a 96-well plate with *S. aureus* SH1000 in MH media and *C. albicans* SAH 1.1 in LB media. An initial OD_{600} of 0.1 (~5×10^5 CFU/mL) was used for the cell cultures prior to incubation. Cultures were treated with **poly-1-Na** (16 µg/ml) in combination with different concentrations of dipyridyl (12.5-400 µM), paraquat (0.125-4 mM), or SDS (0.00125-0.04%). Plates were incubated at 37°C for 20 hours for *S. aureus* and 48 hours for *C. albicans*. Controls included untreated cells and single-agent treatments including for **poly-1-Na** for each biological replicate. The OD_{600} was measured using a FLUOstar Omega microplate reader. Data were analysed in GraphPad Prism (10.4.1) with a nonlinear regression to fit a dose-response curve. Data represents mean ± SD of n = 3 biological replicates in each assay.

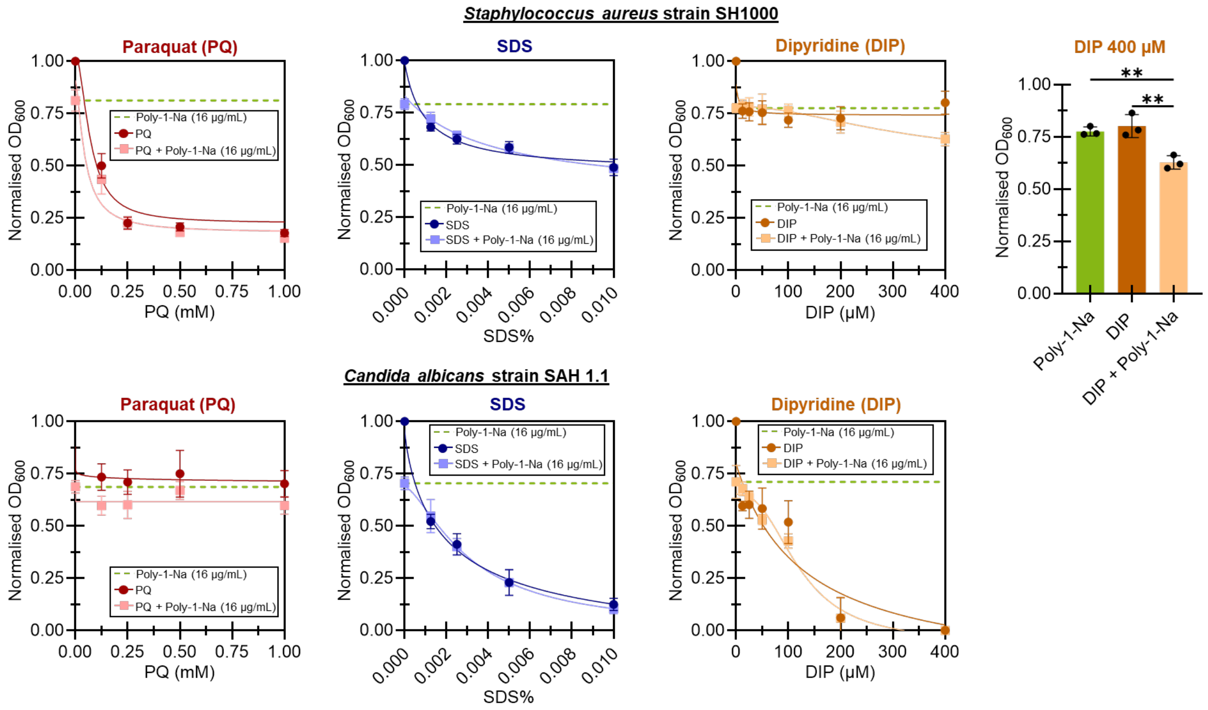

Normalised absorbance of cultures at 600 nm, in the presence of **poly-1-Na** (16 µg/ml) (green dotted line) and dipyridyl (DIP) (12.5-400 µM), paraquat (0.125-4 mM), and SDS (0.00125-0.04%) alone and in combination with **poly-1-Na** (16 µg/ml) during a 20-hour incubation period at 37 °C for *S. aureus* (SH1000) and a 48-hour incubation period at 37 °C for *C. albicans* (SAH 1.1). Data points represent the mean of three biological replicates (n = 3), and error bars indicate the standard error of the mean. The solid line corresponds to a non-linear regression fit (inhibitor vs. response, variable slope) performed in GraphPad Prism.

Effect of poly-1-Na on *Candida albicans* morphology

Apparent hyphal length analysis was performed using *C. albicans* strain SAH 1.1 cultured in LB media and treated with sub-lethal concentrations of **poly-1-Na** (0-32 µg/mL). An initial OD_{600} of 0.1 (~5 × 10^5 CFU/mL) was used for the cell cultures prior to incubation. Treated cultures were incubated at 37°C for 24 hours. After incubation, the OD_{600} was measured using a FLUOstar Omega microplate reader. A 5 µL aliquot of each culture was deposited onto a microscope slide and analysed immidetly. Samples were imaged using an Olympus BX53 microscope equipped with a 10x objective to enable measurement of extended hyphal filaments. Higher-resolution images presented in the manuscript were acquired using a 40x objective.

Apparent hyphal length was measured using ImageJ software following calibration of the image scale. Hyphae that were overlapping were excluded from analysis. Individual hyphae were traced using the segmented line tool, and lengths were recorded in µm. For each concentration of **poly-1-Na** (0, 8, 16, and 32 µg/mL), three biological replicates were performed, with atleast 15 hyphae measured per biological replicate from randomly selected fields of view. Data represents mean ± SD of (n = 3 biological replicates). Statistical analysis was done in GraphPad Prism using ordinary one-way ANOVA with multiple comparisons.

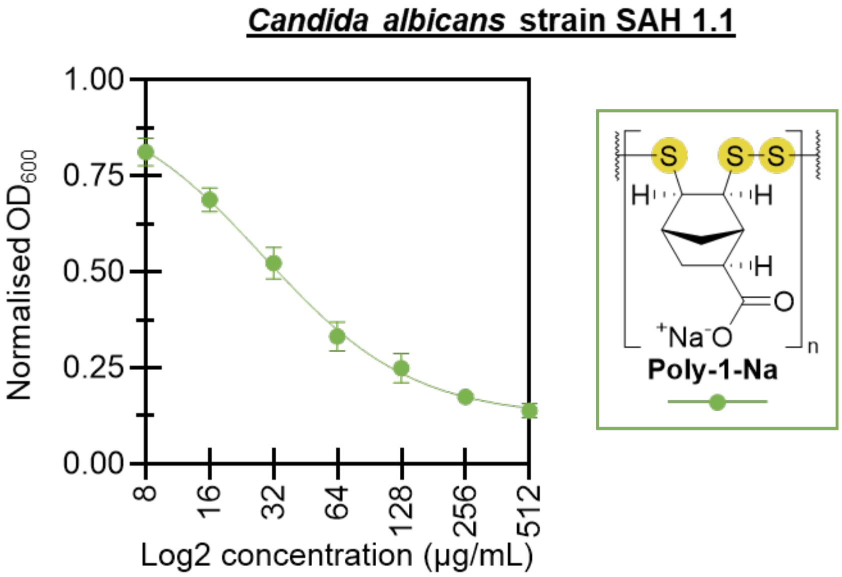

Normalised absorbance of cultures at 600 nm, in the presence of **poly-1-Na** between 8 µg/ml and 512 µg/ml during a 24-hour incubation period at 37 °C. Data points represent the mean of three biological replicates (n = 3), and error bars indicate the standard error of the mean. The solid line corresponds to a non-linear regression fit (inhibitor vs. response, variable slope) performed in GraphPad Prism.

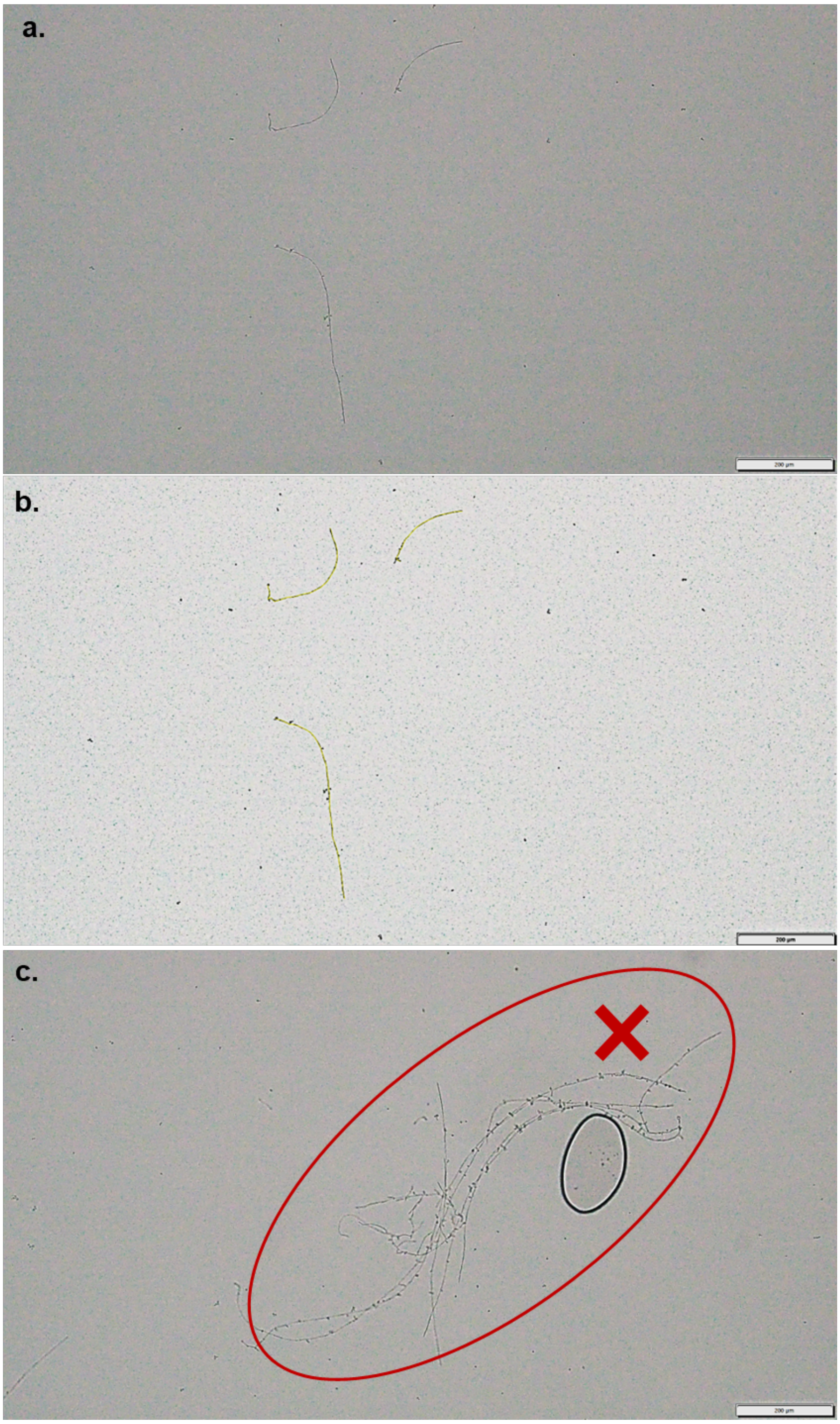

a. Microscope image of C. albicans using a 10x objective. **b.** Image processed in ImageJ. Yellow lines indicate the traced hyphae using the segmented line tool. **c**. Example of overlapping hyphea which were not included in anlaysis.

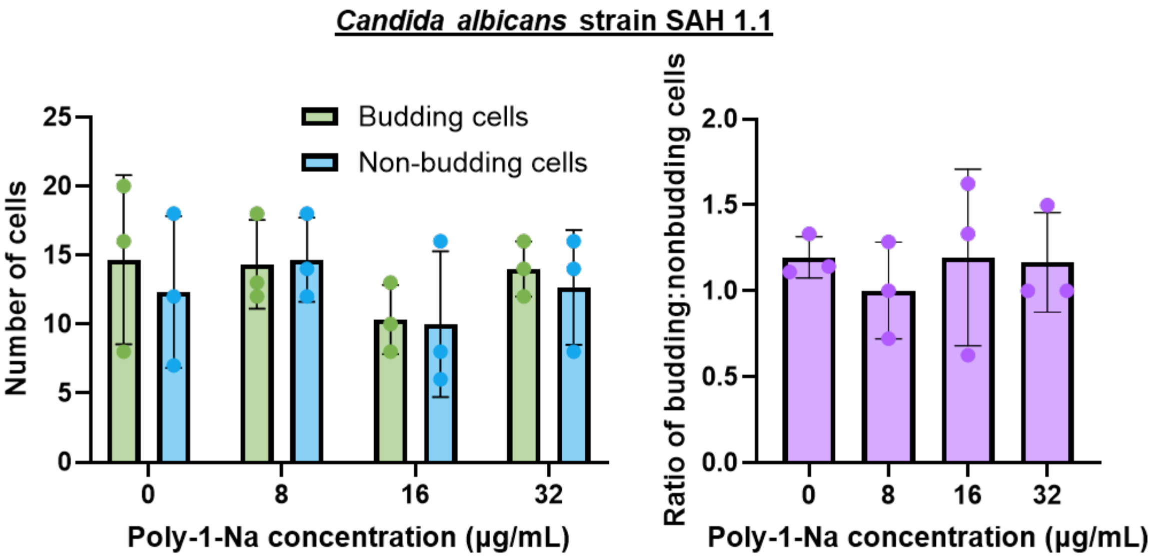

Number of budding and non-budding (left) and ratio of budding: non-bidding (right) *C. albicans* yeast cells in the presence of **poly-1-Na** between 8 μg/ml and 32 μg/ml during a 24-hour incubation period at 37 °C. Data points represent the mean of three biological replicates (n = 3), and error bars indicate the standard error of the mean. Cells were counted from from randomly selected fields of view. No significant difference in budding vs. non-budding yeast cells were observed.

Effect of poly-1-Na on *Staphylococcus aureus* plasma membrane

Membrane integrity of *S. aureus* strain SH1000 treated with poly-1-Na using SYTOX Green stain and flow cytometry. *S. aureus* cultures were grown in MH media to and OD_{600} of 0.5. The cultures were then diluted to an OD_{600} of 0.1 (~5 × 10^5 CFU/mL) and treated with **poly-1-Na** (0, 8, 16, and 32 µg/mL) at 37°C for 2 hours. After incubation, the OD_{600} was measured using a FLUOstar Omega microplate reader. Cells were washed in PBS to remove residual compounds and media. SYTOX green was added to a to a final concentration of 5 mM. Samples were incubated at room temperature in the dark for 15 minutes before analysis.

Prior to analysis, samples were diluted with PBS to a final volume of 0.5 mL and analysed using flow cytometry (Attune NxT) equipped with a 488 nm excitation laser. Controls included unstained cells and ethanol treated cells (dead). Data was analysed by gating cell population to exclude debris. The live fluorescent peak was gated, and its X peak was used to assess membrane integrity. Data represents mean ± SD of (n = 3 biological replicates). Statistical analysis was done in GraphPad Prism using ordinary one-way ANOVA with multiple comparisons.

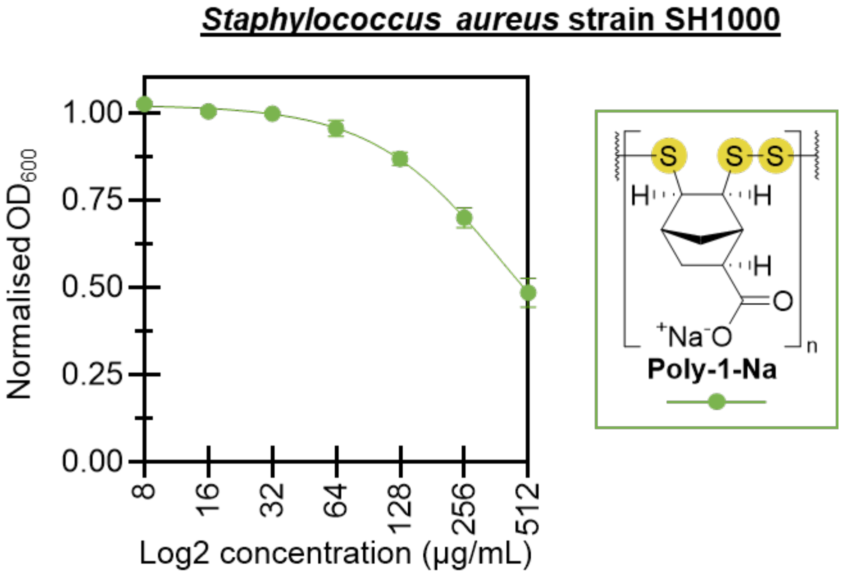

Normalised absorbance of cultures at 600 nm, in the presence of **poly-1-Na** between 8 µg/ml and 512 µg/ml during a 2-hour incubation period at 37 °C. Data points represent the mean of three biological replicates (n = 3), and error bars indicate the standard error of the mean. The solid line corresponds to a non-linear regression fit (inhibitor vs. response, variable slope) performed in GraphPad Prism.

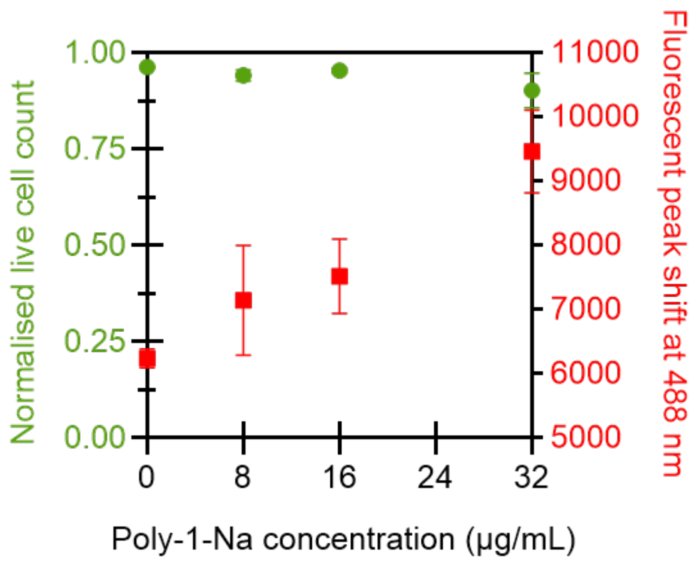

Normalised live cell counts and shift in fluorecent peak of live *S. aureus* cells in the presence of **poly-1-Na** (0, 8, 16, 32 μg/mL). Data points represent the mean of three biological replicates (n = 3), and error bars indicate the standard error of the mean.

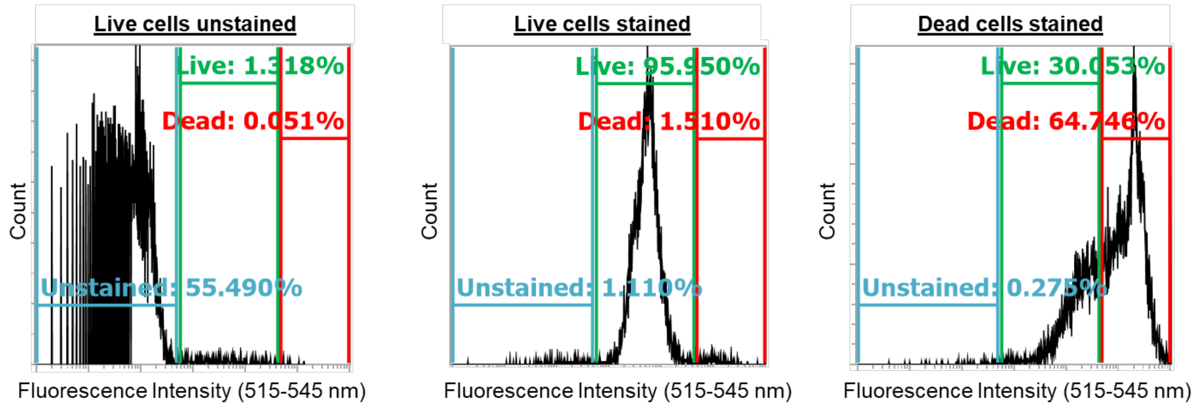

Example fluorescence spectrum at 488 nm excitation and 515-545 nm emission for live unstained cells (left), live stained cells (middle), and dead stained cells (right) with gates.

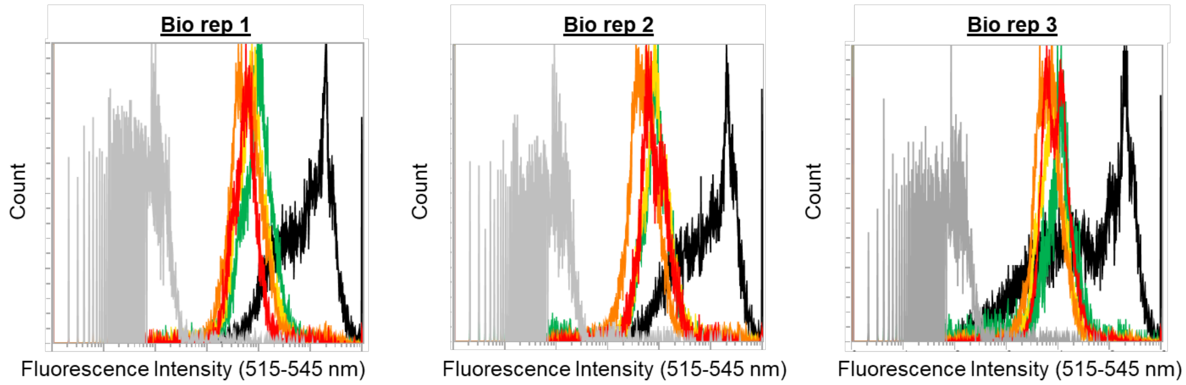

Fluorescence spectrum at 488 nm excitation and 515-545 nm emission for all samples.

Assessment of HepG2 cell viability

Methods:

An MTT Assay (Abcam, ab211091) was used to assess the cell viability/cytotoxicity of **poly-1-Na** and **1-Na**. An MTT assay's principle is the enzymatic reduction of the yellow MTT substrate by mitochondria in active cells into purple formazan crystals. HepG2 cells were maintained in MEM (Gibco, cat. 11095080) supplemented with 10% FBS (Gibco, cat. 10099141), 100 units/mL Penicillin/100 µg/mL Streptomycin (Gibco, cat. 15140122), 1X GlutaMAX (Gibco, cat. 35050061). Cells were seeded at approximately 10^4 cells/well in a 96-well plate. The following day the test compound was added into the culture media at the relevant concentrations and cells were cultured for 24 hrs. Brightfield images were obtained at 20x magnification using ZEISS Primovert microscope equipped with ZEISS Axiocam 208 color camera using the Labscope software. An MTT Assay (Abcam, ab211091) was performed to assess the cell viability/cytotoxicity. Accordingly, growth media was removed and discarded, and then 50 µL of serum free media and 50 µL of the MTT solution were added to each well and incubated at 37°C for 3 hours. 150 µL of the MTT solvent was added to each well and incubated at room temperature, in the dark, with shaking for 15 minutes. Any precipitate formed was resuspended by pipetting and absorbance at 590 nm was then measured (SpectraMax iD5). Assay results were independently replicated (n=2). Controls included no drug (100% viability), 0.2% Triton X-100 treated (0% viability) and media alone (background absorbance). Corrected absorbance readings were calculated for each of the replicate wells using the mean absorbance of the culture medium. Cell viability was calculated as follows

$$Cell\ viability\ (\%) = \frac{A_{sample\ corrected}}{A_{control\ corrected}} * 100$$

Data were analysed in GraphPad Prism (10.4.1) with a nonlinear regression to fit a dose-response curve. Data represents mean ± SD of n = 3 technical replicates in each assay.

Results:

Cytotoxicity of poly-1-Na and 1-Na. The liver cell line HepG2 was incubated for 24 hrs with each compound from 1-256 µg/ml. Visual impact on cell morphology, as assessed by brightfield microscopy, was not observed at <64 µg/ml for **poly-1-Na**. Quantitative assessment of cell viability was determined by MTT assay, with reduced cell viability at 128 and 256 µg/ml and a calculated 50% cytotoxic concentration (CC_{50}) of 147.4 µg/mL for poly-1-Na, while 1-Na had a CC_{50} of 153.4 µg/mL. CC_{50} values were determined using GraphPad Prism, data were analysed with a non-linear regression to fit a dose-response curve. Values represent mean ± SD, n=3.

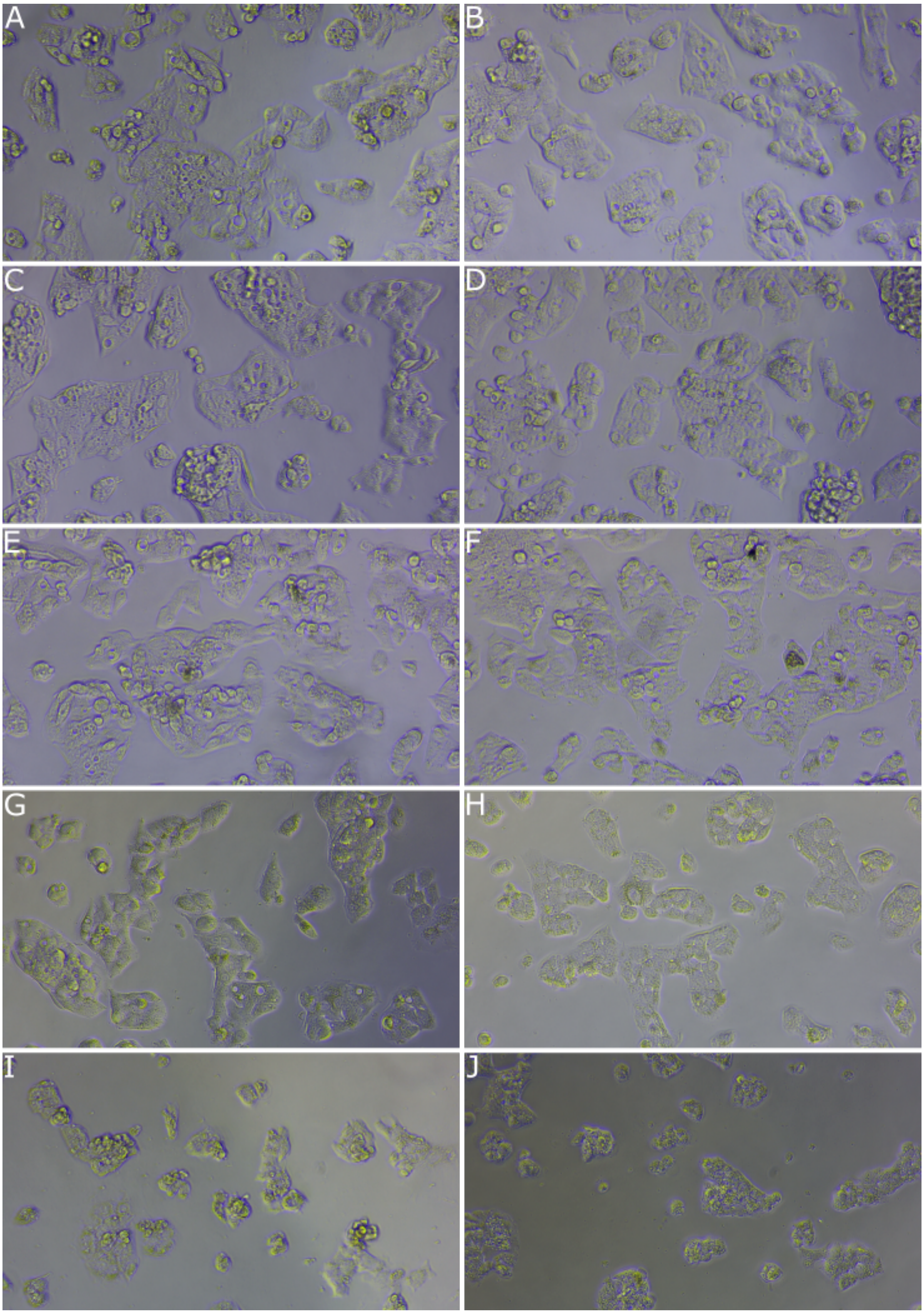

Morphological impact of **poly-1-Na** on HepG2 cells. Cells were cultured for 24h with increasing concentrations of **poly-1-Na** and cell morphology analysed by bright field microscopy. Representative images are shown (20x magnification, ZEISS Primovert with ZEISS Axiocam and Labscope software). A = Media only, B = 1 μg/mL, C = 2 μg/mL, D = 4 μg/mL, E = 8 μg/mL, F = 16 μg/mL, G = 32 μg/mL, H = 64 μg/mL, I = 128 μg/mL, J = 256 μg/mL. Morphological rounding and reduced cell confluency were observed in panels I and J, compared to untreated cells (panel A).

References

1. H. D. Patel, A. D. Tikoalu, J. N. Smith, Z. Pei, R. Shapter, S. J. Tonkin, P. Yan, W. M. Bloch, M. R. Johnston, J. R. Harmer, C. T. Gibson, M. V. Perkins, T. Hasell, M. L. Coote, Z. Jia, J. M. Chalker, *ChemRxiv*, 2025, DOI: 10.26434/chemrxiv-2025-fnhdd
2. F. C. Tenover, R. V. Goering, *J. Antimicrob. Chemother.* 2009, **64**, 441-446.
3. W. A. Fonzi, M. Y. Irwin, *Genetics* 1993, **134,** 717-728.
4. S. Poulain, S. Julien, E. Duñach, *Tetrahedron Lett.* 2005, **46**, 7077-7079.
5. J. M. M. Pople, T. P. Nicholls, L. N. Pham, W. M. Bloch, L. S. Lisboa, M. V. Perkins, C. T. Gibson, M. L. Coote, Z. Jia and J. M. Chalker, *J. Am. Chem. Soc.*, 2023, **145**, 11798–11810.
6. A. A. Miles, S. S. Misra, J. O. Irwin, *J. Hyg.* 1938, **38**, 732-749.
7. A. J. Hedges, *Int. J. Food Microbiol.* 2002, **76**, 207-214.
8. L. J. Bock, C. K. Hind, J. M. Sutton, M. E. Wand, *Lett. Appl. Microbiol.* 2018, **66**, 368-377.
